# Interplay between Subthreshold Oscillations and Depressing Synapses in Single Neurons

**DOI:** 10.1371/journal.pone.0145830

**Published:** 2016-01-05

**Authors:** Roberto Latorre, Joaquín J. Torres, Pablo Varona

**Affiliations:** 1 Grupo de Neurocomputación Biológica, Dpto. de Ingeniería Informática, Escuela Politécnica Superior, Universidad Autónoma de Madrid, 28049, Madrid, Spain; 2 Departamento de Electromagnetismo y Física de la Materia, and Institute Carlos I for Theoretical and Computational Physics, University of Granada, Granada, Spain; Plymouth University, UNITED KINGDOM

## Abstract

In this paper we analyze the interplay between the subthreshold oscillations of a single neuron conductance-based model and the short-term plasticity of a dynamic synapse with a depressing mechanism. In previous research, the computational properties of subthreshold oscillations and dynamic synapses have been studied separately. Our results show that dynamic synapses can influence different aspects of the dynamics of neuronal subthreshold oscillations. Factors such as maximum hyperpolarization level, oscillation amplitude and frequency or the resulting firing threshold are modulated by synaptic depression, which can even make subthreshold oscillations disappear. This influence reshapes the postsynaptic neuron’s resonant properties arising from subthreshold oscillations and leads to specific input/output relations. We also study the neuron’s response to another simultaneous input in the context of this modulation, and show a distinct contextual processing as a function of the depression, in particular for detection of signals through weak synapses. Intrinsic oscillations dynamics can be combined with the characteristic time scale of the modulatory input received by a dynamic synapse to build cost-effective cell/channel-specific information discrimination mechanisms, beyond simple resonances. In this regard, we discuss the functional implications of synaptic depression modulation on intrinsic subthreshold dynamics.

## Introduction

Subthreshold oscillations can be observed in many neuron types and have been proposed to participate in distinct information processing mechanisms both at the single neuron and network levels [1–4]. On the one hand, there is a well-established association between resonance phenomena and membrane potential subthreshold oscillations. On the other hand, the interplay between the intrinsic dynamics that shape the subthreshold oscillations and incoming input with a specific temporal structure can give rise to information discrimination properties in single neurons [5, 6].

Subthreshold oscillations are present in a large variety of cell types in the nervous system and possibly arise from different subcellular and network origins [7–12]. For example, in the dorsal column nuclei (DCN), subthreshold oscillations rely on the interplay of a persistent sodium current and a non-inactivating TEA-sensitive outward current [13], which are thought to underlie the rhythmic activity observed in the DCN. In the neurons of the inferior olive, coordinated subthreshold oscillations act as a timing device to gate inputs [7]. These oscillations require the presence of a low-voltage activated calcium current. In slice recordings, impedance measurements show that all olivary neurons display resonance even if they do not oscillate [7]. In cortical interneurons, subthreshold oscillations have been attributed to an alternating activation of persistent sodium and delayed rectifier channels [10]. In the entorhinal cortex, the joint contribution of near-threshold currents underlies the generation of intrinsic oscillatory activity and associated resonances of stellate cells [14, 15].

In theoretical and computational studies, subthreshold oscillations are seen as a mechanism to implement intrinsic memory and to build preferred input/output relationships, from single neuron resonance phenomena [2, 3, 16] to network spike sequence generation and detection [17, 18]. The basic principles that build up the computational properties of subthreshold oscillations are related to the combination of slow and fast dynamics. The interplay between different time scales gives rise to short-term memory mechanisms and history-dependent information processing [6, 19].

The operating regime of a neuron and its associated input/output transformation is determined by both the pre- and post-synaptic dynamics (e.g., see [19–21]). In the context of neuronal input, many experimental studies have reported that the transmission of signals through synapses is affected by recent presynaptic activity in such a way that, during repetitive stimulation, a postsynaptic response can decrease (*synaptic depression*) or increase (*synaptic facilitation*) at short time scales [22–24]. Several examples have been described in cortical synapses where, after long-term potentiation (LTP), the synaptic response does not increase uniformly. During repetitive presynaptic inputs, the amplitude of the initial postsynaptic potential is potentiated whereas the steady-state synaptic response is unaffected by LTP [25]. This type of short-term synaptic plasticity has its origin in the complex dynamics of release, transmission and recycling of neurotransmitter molecules at synaptic boutons [26]. Synaptic depression occurs when presynaptic high-frequency spikes do not allow an efficient recovering at short time scales of the available neurotransmitters near the cell membrane [26, 27]. This causes a decrease of the postsynaptic response for successive presynaptic action potentials. Synaptic depression can also be caused by other mechanisms such as feedback activation of presynaptic receptors and by postsynaptic processes as receptor desensitization [28]. Synaptic facilitation, on the other hand, is a consequence of the existence of *residual* cytosolic calcium—which remains inside the synaptic boutons after the arrival of the firsts action potentials. This excess of calcium favors the release of more neurotransmitter vesicles for the next arriving action potential since release probability increases with calcium concentration [29]. The increase in released neurotransmitters causes, then, a potentiation of the postsynaptic response or synaptic facilitation.

The last decade has witnessed several studies on the profound consequences of short-term synaptic plasticity on both information transmission by individual neurons as well as on network functioning and behavior (for recent reviews see [24, 30, 31]). For instance, in feed-forward networks activity-dependent synapses act as nonlinear filters in supervised learning paradigms [32], being able to extract statistically significant features from noisy and variable temporal patterns [33]. In recurrent networks, populations of excitatory neurons with depressing synapses exhibit complex regimes of activity [34–38], such as short intervals of highly synchronous activity (population bursts) intermittent with long periods of asynchronous activity, as is observed in neurons throughout the cortex [38]. Short-term synaptic plasticity has also been proposed to shape population dynamics in hippocampus place cells [39]. Synaptic depression may serve as a mechanism for rhythmic activity and central pattern generation [36, 40]. Also, studies with rate models have reported the importance of dynamic synapses in the emergence of persistent activity after removal of stimulus as a base for working memory [41], which could arise by synaptic facilitation mediated by residual calcium [42].

All these phenomena have stimulated much research to elucidate the effect and possible functional role of short-term synaptic plasticity. Synaptic depression induces different non-equilibrium phases in attractor networks depicting high sensitivity to changing stimuli, which can result in the appearance of dynamical memories [43–47]. This dynamical behavior has been associated [48] to empirically observed transitions between states of high activity (Up states) and low activity (Down states) in the mammalian cortex [49, 50]. A recent study [51] characterized the interplay between synaptic depression and the hyperpolarizing potassium current, which has an important role in the termination of the up state [52].

The enhanced sensibility of neural networks to external stimuli due to dynamic synapses provides a controlled mechanism to efficiently process weak signals in a background of noisy activity. In fact, short-term synaptic plasticity together with nonlinear mechanisms affecting neuron excitability can induce efficient signal detection at different noise levels [53–55].

All these studies illustrate that single neuron and network dynamics are highly modulated by dynamic synapses. However, so far the computational properties of subthreshold oscillations and dynamic synapses have been addressed separately. The distinct time scales, the resonance properties and the excitability that arise from the combination of intrinsic subthreshold oscillations and the modulatory input of short-term plasticity can be studied both theoretically and experimentally. In this paper we focus on the interplay between subthreshold oscillations and depressing synapses using a detailed biophysical cell model and multiple synaptic channels. We emphasize the computational properties in the form of input/output preferences that arise from combining the time constants of intrinsic and synaptic dynamics at the single neuron level.

## Methods

### Neuron Model

The individual dynamics of the neuron receiving the different stimuli analyzed in our study was modeled with a Hodgkin-Huxley type formalism proposed in [18] for an inferior olive cell. The single-compartment model consists of five voltage-dependent ionic currents—a sodium current (*I*_*Na*_), a persistent sodium current, (*I*_*Nap*_), a potassium delayed rectifier current (*I*_*Kd*_), a slow inactivating potassium current (*I*_*Ks*_) and a hyperpolarizing potassium current (*I*_*h*_)—and a leakage current (*I*_*l*_). Formally, the membrane voltage is described by the following equation:

$$\begin{array}{c} C_{m}\frac{dV}{dt}=-\left(I_{Na}+I_{Nap}+I_{Kd}+I_{Ks}+I_{h}+I_{l}+I_{syn}\right) \end{array}$$ (1)

where *C*_*m*_ = 1*μF*/*cm*^2^; *I*_*l*_ = *g*_*l*_(*V* − *V*_*l*_) with *g*_*l*_ = 0.1*ms*/*cm*^2^ and *V*_*l*_ = −60*mV*; and *I*_*syn*_ represents the total synaptic current (see below).

The general description of the five active ionic currents considered in the model follows the Hodgkin-Huxley formalism:

$$\begin{array}{c} I_{i}={\bar{g}}_{i}\cdot x^{p}\cdot y^{q}\cdot \left(V-V_{i}\right) \end{array}$$ (2)

where *g*_*i*_ is the maximal conductance of the current, *V* is the membrane potential, *V*_*i*_ is the reversal potential of the current and *x* and *y* are the activation and inactivation variables. Table 1 provides the specific values of these parameters for each ionic current. The activation and inactivation variables, when exist, satisfy the following equations:

$$\begin{array}{c} \frac{dx}{dt}=\frac{x_{\infty }-x}{\tau_{x}},\;\frac{dy}{dt}=\frac{y_{\infty }-y}{\tau_{y}} \end{array}$$ (3)

The steady state and time constants of these dynamical variables for each current are:

*I*_*Na*_:
$m_{\infty }=\frac{\alpha_{m}}{\alpha_{m}+\beta_{m}};\;\;\tau_{m}=\frac{1}{\alpha_{m}+\beta_{m}}$
*α*_*m*_ = 0.1(*V* + 29)/(1 − exp(−0.1(*V* + 29)));   *β*_*m*_ = 4 exp((−*V* − 54)/18);
$h_{\infty }=\frac{\alpha_{h}}{\alpha_{h}+\beta_{h}};\;\;\tau_{h}=\frac{1}{\alpha_{h}+\beta_{h}}$
$\alpha_{h}=1.99\exp\left(\left(-V-43\right)/20\right);\;\;\;\beta_{h}=\frac{28.57}{1+\exp(-0.1(V+13)))};$
*I*_*Nap*_:
*n*_∞_ = Γ(*V*, 51, 5);
*I*_*Kd*_:
$c_{\infty }=\frac{\alpha_{c}}{\alpha_{c}+\beta_{c}};\;\;\tau_{c}=\frac{1}{\alpha_{c}+\beta_{c}};$
*α*_*c*_ = 0.2857(*V* + 33)/(1 − exp(−0.1(*V* + 33)));   *β*_*c*_ = 3.57 exp((−*V* − 43)/80);
*I*_*Ks*_:
*d*_∞_ = Γ(*V*, 34, 6.5);  *τ*_*d*_ = 50*ms*
*e*_∞_ = Γ(−*V*, −65, 6.6);  *τ*_*e*_ = 200. + 220Γ(*V*, 71.6, 6.85);
*f*_∞_ = Γ(−*V*, −65, 6.6);  *τ*_*f*_ = 200. + 3200Γ(*V*, 63.6, 4);
*I*_*h*_:
$t_{\infty }=\Gamma \left(-V,-45,5.5\right);\;\;\tau_{t}=\frac{1}{\left(\exp(-14.59-0.089V)+\exp(-1.87+0.0701V)\right)};$

where $\Gamma \left(X,Y,Z\right)=\frac{1}{1+\exp(-(X+Y)/Z)}$.

**Table 1:** Conductance description, maximal conductances and reverse potential (Eq 2) for the ionic currents of the neuron model.

Current (*μA*/*cm*^2^)	Conductance	${\bar{g}}_{i}$ (*mS*/*cm*^2^)	*V*_*i*_ (*mV*)
*I*_*Na*_	${\bar{g}}_{Na}m_{\infty }^{3}h$	${\bar{g}}_{Na}=52$	*V*_*Na*_ = 55
*I*_*Nap*_	${\bar{g}}_{Nap}n_{\infty }$	${\bar{g}}_{Nap}=0.1$	*V*_*Na*_ = 55
*I*_*Kd*_	${\bar{g}}_{Kd}c^{4}$	${\bar{g}}_{Kd}=20$	*V*_*K*_ = −90
*I*_*Ks*_	${\bar{g}}_{Ks}d\left(0.6e+0.4f\right)$	${\bar{g}}_{Ks}=14$	*V*_*K*_ = −90
*I*_*h*_	${\bar{g}}_{h}t$	${\bar{g}}_{h}=0.1$	*V*_*h*_ = −43

**Fig 1:**
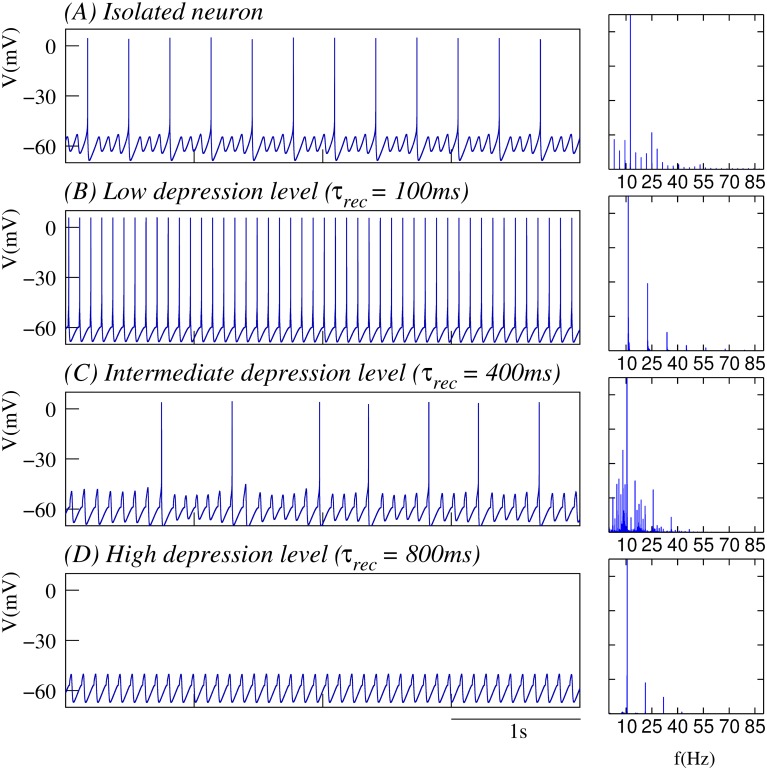
Synaptic depression tunes the resonant response to incoming input. (A) Behavior of the isolated single neuron with subthreshold oscillations and spiking activity. (B-D) Neuron’s response to incoming input delivered at the peak of the subthreshold depolarization as a function of different depression levels. In all cases, the input is received through a dynamic synapse (Eq 4) with *g*_*syn*_ = 0.25*mS*, *τ*_*fac*_ = 0.02*ms*, i.e., a synapse without facilitation, and U=0.45; and different *τ*_*rec*_ values, which define the depression level: low in panel B (*τ*_*rec*_ = 100*ms*), intermediate in panel C (*τ*_*rec*_ = 400*ms*) and high in panel D (*τ*_*rec*_ = 800*ms*). Plots in the right column correspond to the normalized power spectra of the output signal. The stimulation protocol used in these simulations maximizes the resonant response (see main text). Nevertheless, depending on the depression level, the neuron response can go from tonic spiking to pure subthreshold activity without spikes.

The model as defined here generates a stereotyped behavior of subthreshold oscillations and spiking activity shown in Fig 1A.

### Synaptic Model

To describe the synaptic input arriving at the conductance-based neuron, we combine the model for synaptic conductances by Destexhe et al. [56] and the description of dynamic synapse currents by Tsodyks et al. [23, 37]. The synaptic current received by a postsynaptic neuron at a given moment is obtained as a function of the fraction of bound receptors as follows:

$$\begin{array}{c} I_{syn}\left(t\right)=g_{syn}\cdot r\left(t\right)\cdot \left[V_{post}\left(t\right)-E_{syn}\right], \end{array}$$ (4)

where *g*_*syn*_ is the synaptic maximal conductance, *V*_*post*_ the postsynaptic potential, *E*_*syn*_ the synaptic reversal potential (in our case, as we are modeling excitatory connections, *E*_*syn*_ = 0*mV*), and the synaptic conductance variable *r* gives the fraction of open channels in the postsynaptic neuron. As in the original work by Destexhe et al. [56], the kinetics of open channels depend on neurotransmitter concentration in the synaptic cleft, [*T*]. To estimate the value of [*T*], we assume that neurotransmitters are quickly released with the arrival of each presynaptic action potential at a given time *t* = *t*_*release*_ and remain in the synaptic cleft for a time interval Δ*t*, i.e., after this time interval [*T*] = 0*mM* until the next release event. In this work, we assume that Δ*t* = 1*ms* [57, 58]. The original Destexhe et al. approach considers that [*T*] has the same fixed value during Δ*t* for all synaptic events. This results in a constant maximum amplitude in the synaptic response to each incoming action potential, whatever the presynaptic input frequency, which in many cases is not realistic [25, 59]. Here, we consider that [*T*] is frequency dependent which in fact implies a dependence of the synaptic strength on the frequency of the presynaptic neuron activity. To achieve this, we have adapted the Destexhe et al. synaptic conductance model assuming that during the release period:

$$\begin{array}{c} \left[T\right]=\kappa \cdot x\left(t_{release}\right)\cdot u\left(t_{release}\right), \end{array}$$ (5)

where *κ* = 1*mM*; and *x*(*t*) and *u*(*t*) are the fraction of neurotransmitters in the ready releasable pool of the presynaptic neurons and the release probability, respectively, which follow the dynamics:

$$\begin{array}{c} \frac{dx(t)}{dt}=\frac{1-x(t)}{\tau_{rec}}-x\left(t\right)\cdot u\left(t\right)\cdot \delta \left(t-t_{release}\right) \end{array}$$ (6)

$$\begin{array}{c} \frac{du(t)}{dt}=\frac{U-u(t)}{\tau_{fac}}+U\cdot \left[1-u\left(t\right)\right]\cdot \delta \left(t-t_{release}\right). \end{array}$$ (7)

Variables *x* and *u* define, respectively, the so called short-term synaptic depression and facilitation processes [23, 37]. Note that in Eqs 6 and 7, the delta functions indicate that the second terms in the right hand side only contribute when *t* = *t*_*release*_. Here *τ*_*rec*_ and *τ*_*fac*_ are the recovery and the facilitation time constants. In addition, U is the release probability in the absence of facilitation and contributes to determine the depression level (the higher its value, the stronger depression).

Finally, the dynamics of the synaptic conductance variable *r*(*t*) is described by the following equation:

$$\begin{array}{c} \frac{dr(t)}{dt}=\alpha \;\left[T\right]\;(1-r\left(t\right))-\beta \;r\left(t\right). \end{array}$$ (8)

Here *α* and *β* are the forward and backward rate constants for transmitter binding. In all simulations presented in this paper, *α* = 2.0*ms*^−1^ *mM*^−1^ and *β* = 1.0*ms*^−1^. The synaptic current is calculated using the conductance variable *r* as given by Eq 4.

Note that, although in this work we present a study of the interplay between subthreshold oscillations and short-term synaptic depression, a similar analysis can be extended to include also synaptic facilitation competing with synaptic depression processes. The proposed model preserves (see S1 Fig) all relevant features observed in the original Tsodyks-Markram model [23, 37] while providing a dynamic synaptic conductance description.

All equations of our model were numerically solved with a Runge-Kutta6(5) variable step method with a maximum error of 10^−18^.

### Stimulation

**Fig 2:**
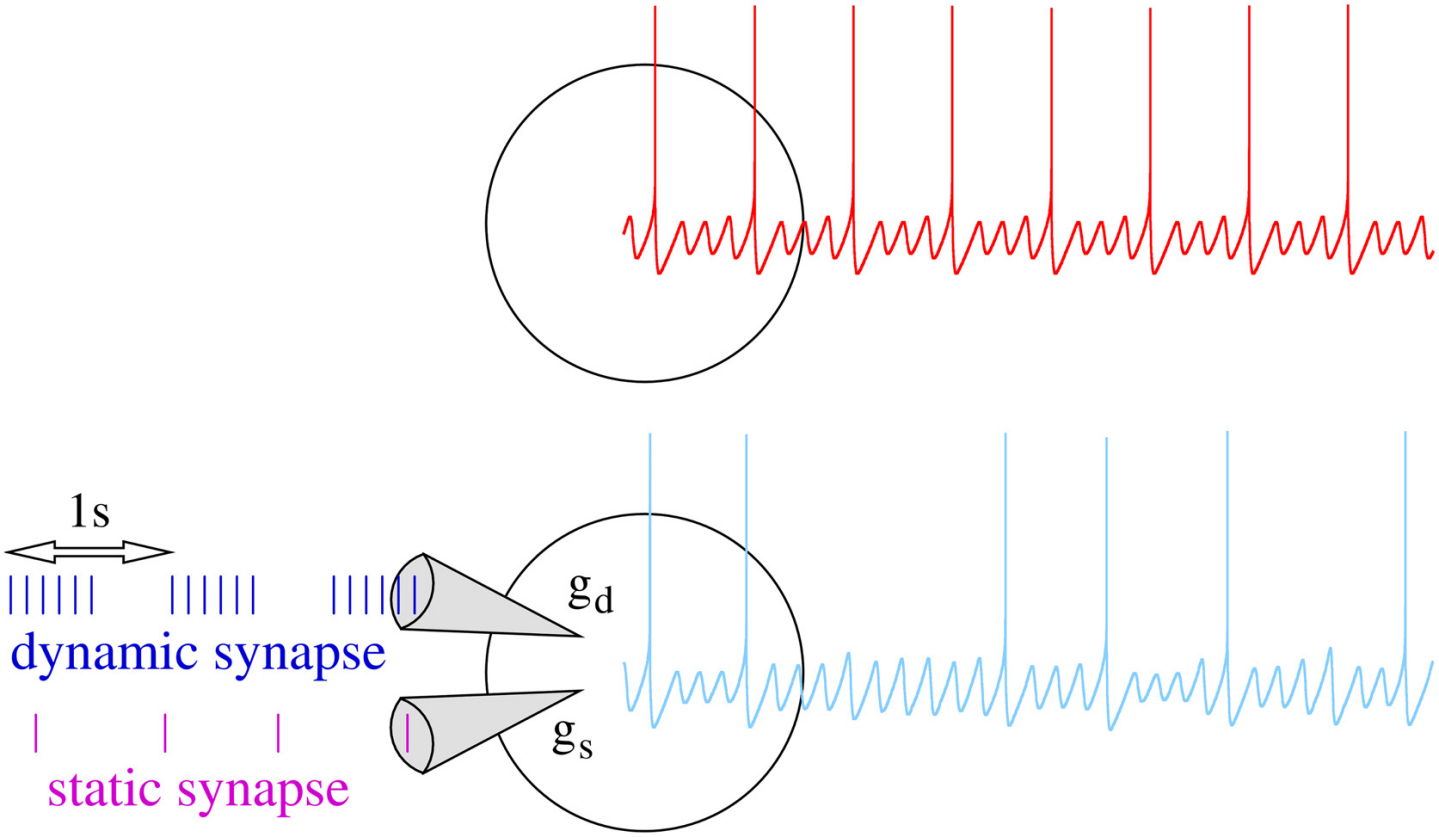
Schematic representation of the experimental setup. Top panel: The isolated neuron generates a characteristic subthreshold oscillation and spiking activity (red trace). Bottom panel: Responses of the neuron to stimuli received through two different input channels are analyzed. The first channel is driven by a dynamic synapse (blue input sequence), i.e., a synapse in which the input current depends on the frequency of the presynaptic activity. Action potentials arriving through the dynamic synapse are grouped in bursts with a regular bursting frequency, while the spike frequency within the burst depends on the specific simulation. Additionally, the neuron can receive a second stimulus through a static synapse (magenta input sequence). In this case, the stimulation consists of individual presynaptic action potentials delivered at a tonic frequency which is a parameter of the experiment.

In a general scenario, neurons can receive thousands of inputs through synapses with different short-term dynamic properties. Here, we consider a minimal setting illustrated in Fig 2 oriented to investigate the input/output relations that arise from the combination of the neuron’s intrinsic subthreshold oscillations and a dynamic synapse. We consider two input channels to the neuron model: a dynamic synapse with short-term synaptic depression and a “static” synapse (i.e., a synapse without depression or facilitation mechanisms). The presence of heterogeneity in synaptic properties has been reported in several neuron types and circuits of the nervous system [60–62]. Our reductionist goal is to analyze (i) the neuron’s response to incoming periodic input delivered through the dynamic synapse considering the modulation of the neuron’s resonant properties by the synaptic depression, and (ii) the response to a simultaneous input received through the second static channel in the context of this modulatory effect.

In the present work, we are interested in the neuron’s history-dependent processing arising from the interaction between the subthreshold oscillation activity and the temporal structure of the inputs. As a simple approach to address this issue, we build the input delivered through the dynamical channel as action potentials grouped into bursts with a regular interburst interval of 1*s* (see blue traces at the bottom panel in Fig 2) and quiescence periods where the neuron dynamics evolves freely. Neural systems such as the inferior olive receive this kind of rhythmic input. We vary the number of spikes per burst from 2 to 10 considering intraburst periodic frequencies from 5*Hz* to 50*Hz* to obtain different depression levels (see S1 Fig) and analyze the dependence of the neuron response on these parameters. Thus, burst duration and quiescence periods depend on these values. The input arriving through the second channel consists of a sequence of spikes with a tonic frequency (magenta traces in schematic representation of Fig 2). Our goal with this second input is to show how it can be distinctly processed depending on the specific modulation of the subthreshold oscillations by the dynamical synapse. We consider different tonic input frequencies to analyze how the synaptic modulation reshapes the resonant properties of the neuron. The specific properties of the inputs received by the neuron will be described in each simulation.

## Results

The response of a neuron with subthreshold oscillations to input arriving through a dynamic synapse can largely depend on the level of synaptic depression. As a first step to characterize the interplay between subthreshold oscillations and short-term synaptic depression, we show in Fig 1 several representative examples of the distinct resonant response as a function of the depression level as specified by the value of *τ*_*rec*_. For comparison, panel A depicts the membrane potential of the isolated neuron and its corresponding power spectra. This panel shows that for the set of neuron parameters considered in this work, the isolated neuron displays a periodic behavior with subthreshold oscillations at ∼ 12.5*Hz* and a ∼ 3*Hz* spiking frequency. Panels B-D illustrate the respond of the neuron to synaptic input delivered when the subthreshold oscillations reach their depolarization peak, thus maximizing the resonant effect at the subthreshold oscillation frequency. The highest response in terms of the spiking frequency is obtained when the depression level is low (see panel B). As the depression level increases (panel C), the spiking frequency diminishes until only subthreshold oscillations are produced in response to incoming input for high depression values (panel D). Panels on the right show the broad power spectra when the neuron displays both subthreshold oscillations and spiking activity, as compared to the sharp peaks observed in the cases when the activity is either pure spiking or pure subthreshold. These power spectra show that synaptic depression does not only affect the spiking activity, but also the subthreshold dynamics. Note the slight decrease in the subthreshold oscillation frequency with increasing values of *τ*_*rec*_. When the stimulus is not delivered at the peak of the subthreshold depolarization, but at the specific timing of a presynaptic input, a modulation of the amplitude and frequency of the oscillations occurs as a function of the depression level. This results in different subthreshold and spiking activity modes, which can implement preferred input/output relations beyond simple resonant responses as we will discuss below.

### Input/output relations are modulated by synaptic depression

#### Shaping subthreshold oscillations and spiking responses by synaptic depression

Synaptic depression decreases the postsynaptic response for successive incoming spikes and this decrease is accentuated for presynaptic spikes arriving at higher frequency (see for instance S1 Fig). As time series in Fig 1 show, this effect reduces the number of action potentials as the level of depression is increased. Our study reveals that the interaction among the multiple time scales involved in the depressing processes and the intrinsic subthreshold dynamics reshapes the resonant properties of the neuron. This leads to the emergence of preferred input-output relations in the form of stereotyped responses to specific input patterns. To expose and characterize these input/output relationships induced by short-term synaptic depression in the neuron, we have built activity maps that illustrate the combined effect of intrinsic and synaptic dynamics. The maps depict the neuron’s mean subthreshold oscillation frequency and mean output spiking frequency as a function of the depression level and the input stimulation frequency through the dynamic channel. Mean data are calculated using a sliding window comprising two stimulation episodes in this channel.

**Fig 3:**
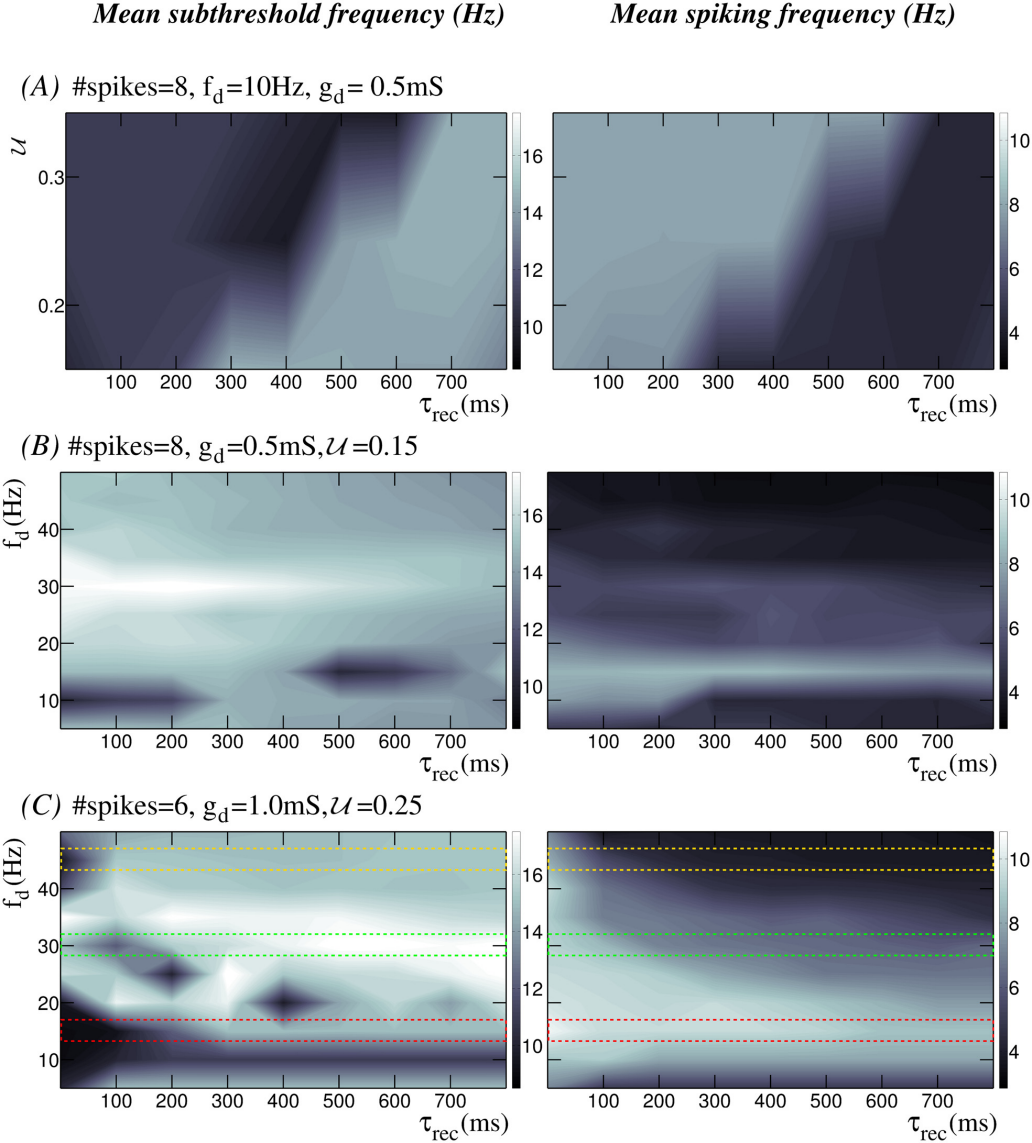
Input/output relations arising from the combination of the subthreshold activity and the short-term depression of a dynamic synapse. Panels display input/output activity maps showing the mean subthreshold oscillation frequency (left column) and the mean output spiking frequency (right column) for different depression levels. #*spikes* and *f*_*d*_ denote the number of spikes per burst and the intraburst stimulation frequency, respectively. (A) Neuron’s response to an input delivered through the dynamic synapse as a function of parameters *τ*_*rec*_ and U which set the depression level. In these maps, *x*-axis ranges from 0.02*ms* to 800*ms* with a step of 100*ms*, and *y*-axis ranges from 0.05 to 0.35 at intervals of 0.05. (B-C) Input/output relations dependence on the intraburst stimulation frequency. Maps are built with a fixed value of U and for different stimulation frequencies delivered through the dynamic synapse (*y*-axes in these panels, with *f*_*d*_ ranging from 5*Hz* to 50*Hz* at intervals of 5*Hz*). Yellow, green and red dashed-rectangles in maps shown in panel C indicate the regions analyzed in detail in Figs 4, 5 and 6, respectively.

We have explored activity maps for a wide range of parameters affecting the synaptic current, i.e., stimulation frequency, number of presynaptic spikes, synaptic weight, U and *τ*_*rec*_. Fig 3 displays three representative examples of these activity maps. Panel A shows the dependence of the neuron’s response on the dynamic synapse parameters shaping the depression level (*τ*_*rec*_ and U). Here, the presynaptic stimulus is built by a regular spiking-bursting signal with 8 action potentials per burst and an intraburst spiking frequency of 10*Hz* received through a dynamic synapse with *g*_*d*_ = 0.5*mS*. This choice produces a moderate depression effect. The modulatory effect of synaptic depression on the neuron dynamics can be observed in these maps, which show two clearly distinct regions (light and dark areas in panel A). These maps illustrate a general inverse relationship between subthreshold (left column) and spiking (right column) activity, i.e., the higher the spiking frequency, the lower the subthreshold frequency and vice versa. Due to the nonlinear interplay between the dynamic synapse and the intrinsic neuron dynamics arising from their distinct interacting time scales, there is not a unique response map as a function of the synapse parameters *τ*_*rec*_ and U. For example, when the stimulation frequency is higher or the synaptic conductance is larger, the activity maps can differ from those shown in Fig 3A. However, the modulatory effect of the input/output relations by the depression holds for a large region of the parameter space.

In the maps shown in panels B and C of Fig 3 the value of U is fixed, and the neuron’s response is characterized as a function of the intraburst stimulation frequency delivered through the dynamical channel and its corresponding depression level given by the value of *τ*_*rec*_. In panel B, the neuron receives a stimulus with 8 spikes at different frequencies through a dynamic synapse with *g*_*d*_ = 0.5*mS* and U=0.15. The maps in this panel show multiple input/output relations, both in the subthreshold and in the spiking activity. Considering the time series shown in Fig 1, one could intuitively expect that a high stimulation frequency combined with a large level of short-term depression would lead to a lower spiking frequency response. For example, the activity maps show that for high intraburst input frequencies (35*Hz* or higher), the neuron produces a low-frequency spiking output (between 3.5 and 4.7*Hz*). Nevertheless, beyond the expected effects of synaptic depression, multiple regions in these maps reflect the complex interaction among the distinct time scales shaping the resonant properties of the neuron, which also results in unexpected preferred input/output relations. For instance, maps in Fig 3B (see also S2 Fig) show that although the spiking response to an intraburst input frequency at 15*Hz* is not affected by short-term synaptic plasticity—independently of the *τ*_*rec*_ value this low-frequency stimulus produces a mean spiking response around 8*Hz* –, the output subthreshold response is shaped by the depression level. For values of *τ*_*rec*_ between 500*ms* and 700*ms*, the neuron generates a low-frequency subthreshold response in the range 10–12*Hz*, while for other depression levels the output subthreshold oscillation is around 15*Hz*, i.e., the subthreshold activity follows the incoming stimulus. Another example of synaptically modulated input/output relation is observed in Fig 3B for lower stimulation frequencies (e.g., 10*Hz*). In these cases, the decrease of the postsynaptic response due to depression is weak and, therefore, each incoming spike should in principle be followed by the generation of an output action potential, i.e., the expected spiking response would at least be near to 8*Hz*. Nevertheless, the mean spiking frequency output for these stimuli is around 4.5*Hz*.

Panel C in Fig 3 shows an example of how the activity maps change as a function of the value of other parameters affecting the depressing input currents (cf. maps in panel B). In particular, it shows the activity maps when the neuron receives a stimulus with 6 spikes through a dynamic synapse with *g*_*d*_ = 1.0*mS* and U=0.25. In general, there exist regions in the parameter space with input/output relations like the ones previously described. However, for specific bounded regions, the response in terms of both subthreshold and spiking activity differs. In the following sections, we discuss in detail the regions highlighted with colored dashed-rectangles where the rich interplay between depression and the intrinsic subthreshold dynamics acts as a cost-effective mechanism to selectively change input/output relations, i.e., a mild tuning in the dynamic synaptic modulation can produce a very different neuron output.

#### Dependence on stimulation frequency and depression level

**Fig 4:**
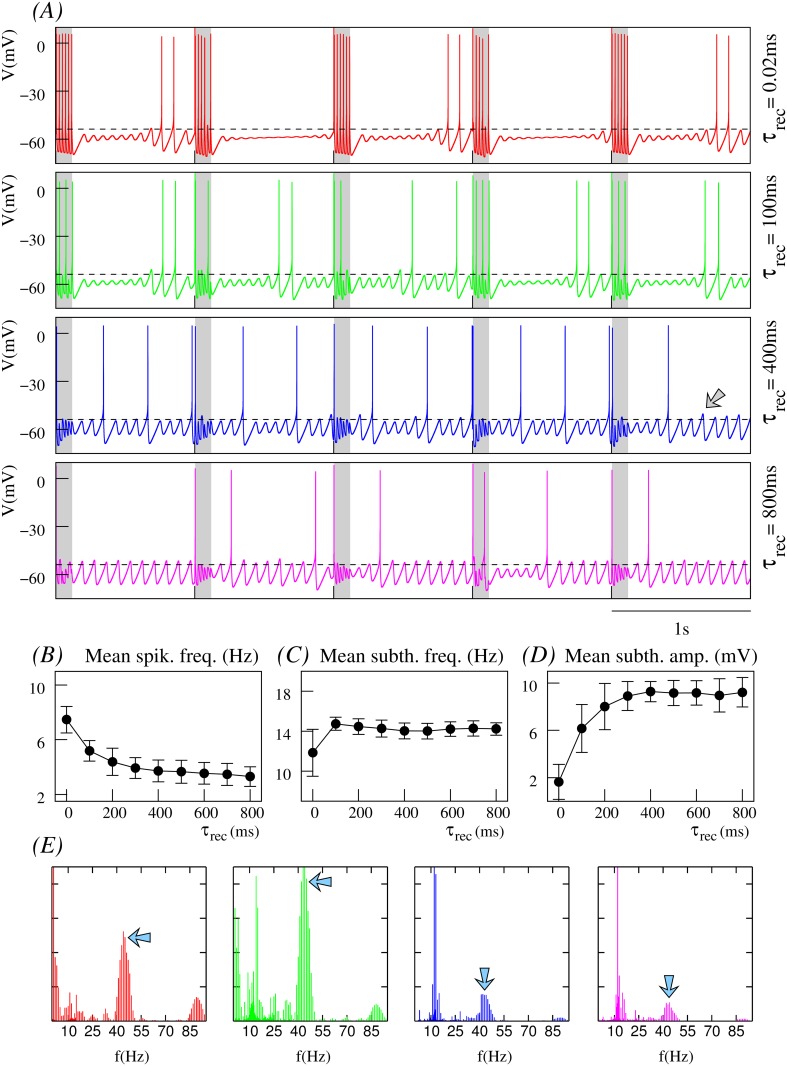
Effect of depression on the neuron dynamics at a high spiking stimulation frequency. The stimuli arrives through a dynamic synapse with *g*_*d*_ = 1.0*mS* and U=0.25. Input consists of sequences of bursts with 6 spikes each delivered at 1*Hz* and with an intraburst spiking frequency of 45*Hz*. This corresponds to the regions of the input/output activity maps highlighted in yellow in Fig 3C. (A) Time series showing the postsynaptic neuron activity for different levels of synaptic depression as indicated by the value of *τ*_*rec*_. Grayed areas correspond to the stimulation periods. Dashed lines provide a fixed voltage reference (firing threshold for *τ*_*rec*_ = 0.02*ms*) to compare firing threshold changes under the effect of synaptic depression. Note that the firing threshold changes for different depression levels, but also dynamically for the same *τ*_*rec*_ value (cf. the spike before the arrow and following oscillations in the blue trace). (B-D) Dependence of mean spiking frequency, mean subthreshold frequency and mean subthreshold amplitude of the postsynaptic neuron on the depression level. Mean data are calculated in 180s time series recorded 10s after the beginning of the stimulation using a sliding window comprising two stimulation episodes in the dynamic channel. (E) Normalized power spectra corresponding to the time series depicted in panel A identified by the same color code. Blue arrows indicate the peak corresponding to the intraburst stimulation frequency.

**Fig 5:**
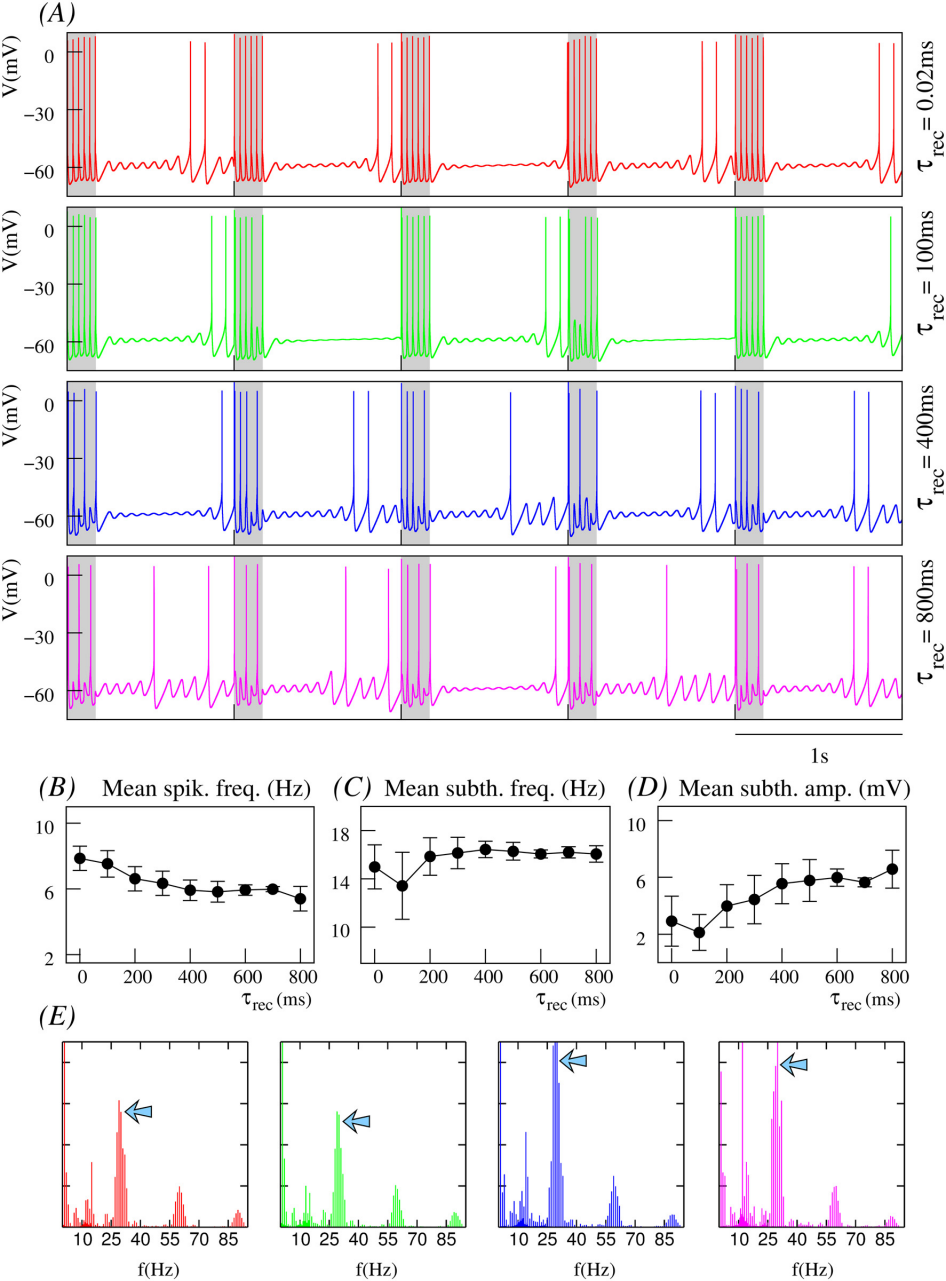
Effect of depression at an intermediate spiking stimulation frequency. Neuron’s response when it receives an equivalent input to the one described in Fig 4, but with an intraburst spiking frequency at 30*Hz* instead of at 45*Hz*—i.e., green regions in the maps of Fig 3C. Red, green, blue and magenta traces correspond to the same depression levels as in Fig 4. Blue arrows in the power spectra indicate the stimulation frequency.

**Fig 6:**
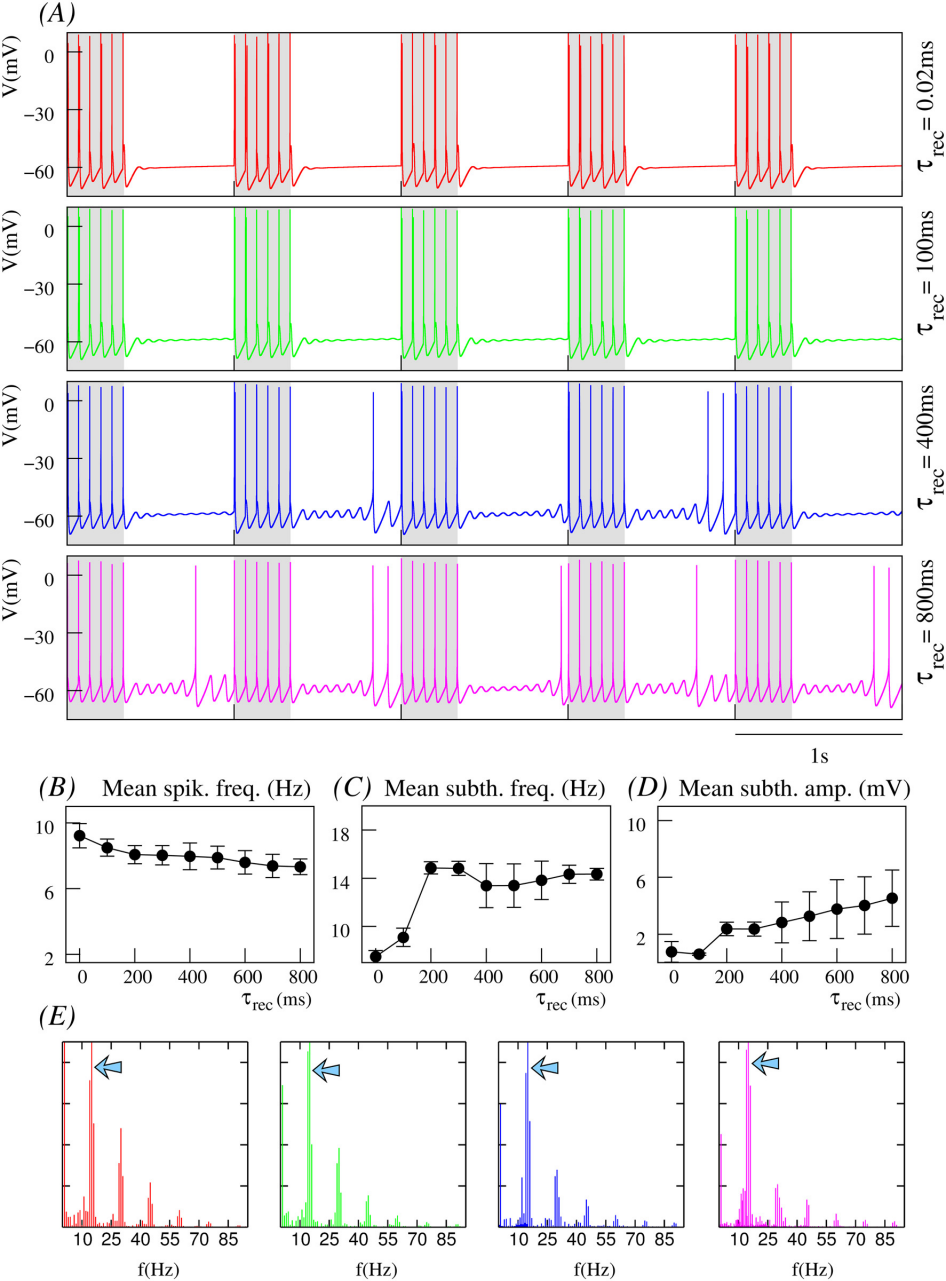
Effect of depression at low spiking stimulation frequency. The figure shows the same analysis as in Figs 4 and 5, but when the intraburst spiking frequency of the incoming signal is 15*Hz* (red region in the input/output activity maps of Fig 3C).

For a better understanding of the origin of the modulatory effect of short-term synaptic depression in the neuron dynamics, it is convenient to analyze the evolution of membrane potential when the neuron receives the same stimulus through synapses with different depression levels (i.e., by fixing the intraburst stimulation frequency in the maps). Figs 4, 5 and 6 show this analysis for three regions highlighted in yellow, green and red in Fig 3C. These regions correspond to sequences of presynaptic bursts of spikes arriving through the dynamic synapse with a high, intermediate and low intraburst spiking frequency (45*Hz*, 30*Hz* and 15*Hz*, respectively). Time series in these figures indicate that short-term synaptic depression reduces the number of action potentials during the stimulation period (shadowed gray intervals in panels A) as the level of depression is increased (cf. times series from top to bottom in panel A in these figures). In general (see the activity maps depicted in Fig 3B and 3C), the higher the depression level, the lower the postsynaptic spiking frequency. This characteristic effect is better appreciated when the postsynaptic neuron receives high-frequency stimuli (e.g., 45*Hz*), through dynamic synapses with relatively large maximal synaptic conductance (*g*_*d*_ = 1.0*mS*), as it is depicted in Fig 4. Thus, in the absence of synaptic depression or for a low depression level (*τ*_*rec*_ small), the neuron follows or nearly follows the incoming stimulus during the stimulation periods. However, as the level of depression increases, the postsynaptic neuron fails to generate a postsynaptic action potential immediately after the arrival of each presynaptic spike within the stimulation interval. The result is that the mean spiking frequency of the postsynaptic neuron decreases with the depression level until it reaches a stable regime (see Fig 4B). Failure in action potential generation following the arrival of presynaptic spikes decreases when the stimulation frequency is lowered, as depicted in the time series shown in Fig 5A for a 30*Hz* stimulation, and even vanishes, for example, for the case of the 15*Hz* stimulation shown in Fig 6A, where the postsynaptic neuron always fires following the incoming spikes during the stimulation period regardless of the depression level. In all cases, when the level of depression is high, the neuron can fire action potentials sporadically during the interstimulation intervals. Note that the mean spiking frequency grows as the intraburst stimulation frequency decreases (cf. Figs 4B, 5B and 6B).

In a postsynaptic neuron with intrinsic subthreshold oscillations, synaptic depression not only affects the spiking activity but also the subthreshold dynamics, as shown in Fig 3. The trend observed in our simulations is that, for low depression levels, the presynaptic stimuli induce subthreshold oscillations with small mean amplitude after the stimulation period. In these cases, the synaptic input can even make the subthreshold activity disappear (e.g., see time series at the top panels of Fig 6A). In general, we observe that there is a monotonic increase of the mean amplitude of the subthreshold oscillations as the level of depression is increased, as depicted in Figs 4D, 5D and 6D, and that this effect is larger for higher stimulation frequencies.

Another relevant effect of synaptic depression on subthreshold dynamics is observed during the stimulation period for high stimulation frequencies. Synaptic depression can induce the generation of subthreshold oscillations with properties that significantly differ from those associated to the intrinsic neuron dynamics in the absence of stimulation (e.g., see the faster subthreshold oscillations in the blue and magenta time series in Fig 4A). These altered subthreshold oscillations are a consequence of postsynaptic action potential generation failures after the arrival of the presynaptic spikes during the stimulation period. When subthreshold oscillations are present, the modulatory effect of synaptic depression transiently affects the neuron dynamics during the stimulation period. Once the stimulation is over, the intrinsic dynamics recovers and the mean subthreshold frequency tends to be around 14–16*Hz* (see Figs 4C, 5C and 6C).

These results reveal the large flexibility provided by the different time scales that participate in the resonant processes to produce the neuron’s response. The interaction of synaptic depression on both subthreshold and spiking dynamics results in different firing thresholds. The fixed reference depicted as a dashed line in Fig 4A allows to note the firing threshold change as a function of the depression level. This reference corresponds to the fixed spike threshold without depression. Note that the resulting firing threshold is dynamic as illustrated by the arrow in the blue trace (the same effect can also be observed in the time series displayed in Figs 5 and 6). The dynamic firing threshold variability underlies all input/output relations shown in Fig 3.

To better quantify relevant emergent phenomena in our study we have computed also the power spectra of times series depicted in Figs 4A, 5A and 6A. These power spectra are shown in Figs 4E, 5E and 6E, respectively. On one hand, one clearly distinguishes the corresponding stimulation frequencies which are the main frequency components (blue arrows) for low synaptic depression levels. On the other hand, power spectra also depict the common intrinsic subthreshold oscillation frequency which becomes the main frequency component for high synaptic depression levels (cf. power spectra depicted in green and magenta). Note that this frequency corresponds to the intrinsic subthreshold oscillation when the neuron is not stimulated (around 12*Hz* and 13*Hz*, cf. power spectra in Fig 1A). In the simulations with the lower stimulation frequency considered in the analyses presented in Figs 4, 5 and 6—corresponding to 15*Hz* –, both the stimulation frequency and subthreshold oscillation frequency are nearly the same. Moreover, due to the relatively small value of the stimulation frequency, there is not a clear effect of synaptic depression reflected in the power spectrum of the corresponding times series (see Fig 6E).

### Response to additional input in the context of the depression modulation

In Fig 3 we have discussed several representative examples of activity maps illustrating the existence of input/output relations induced by short-term synaptic depression. These preferences can largely shape the response to a standardized input delivered through a second *static* channel.

**Fig 7:**
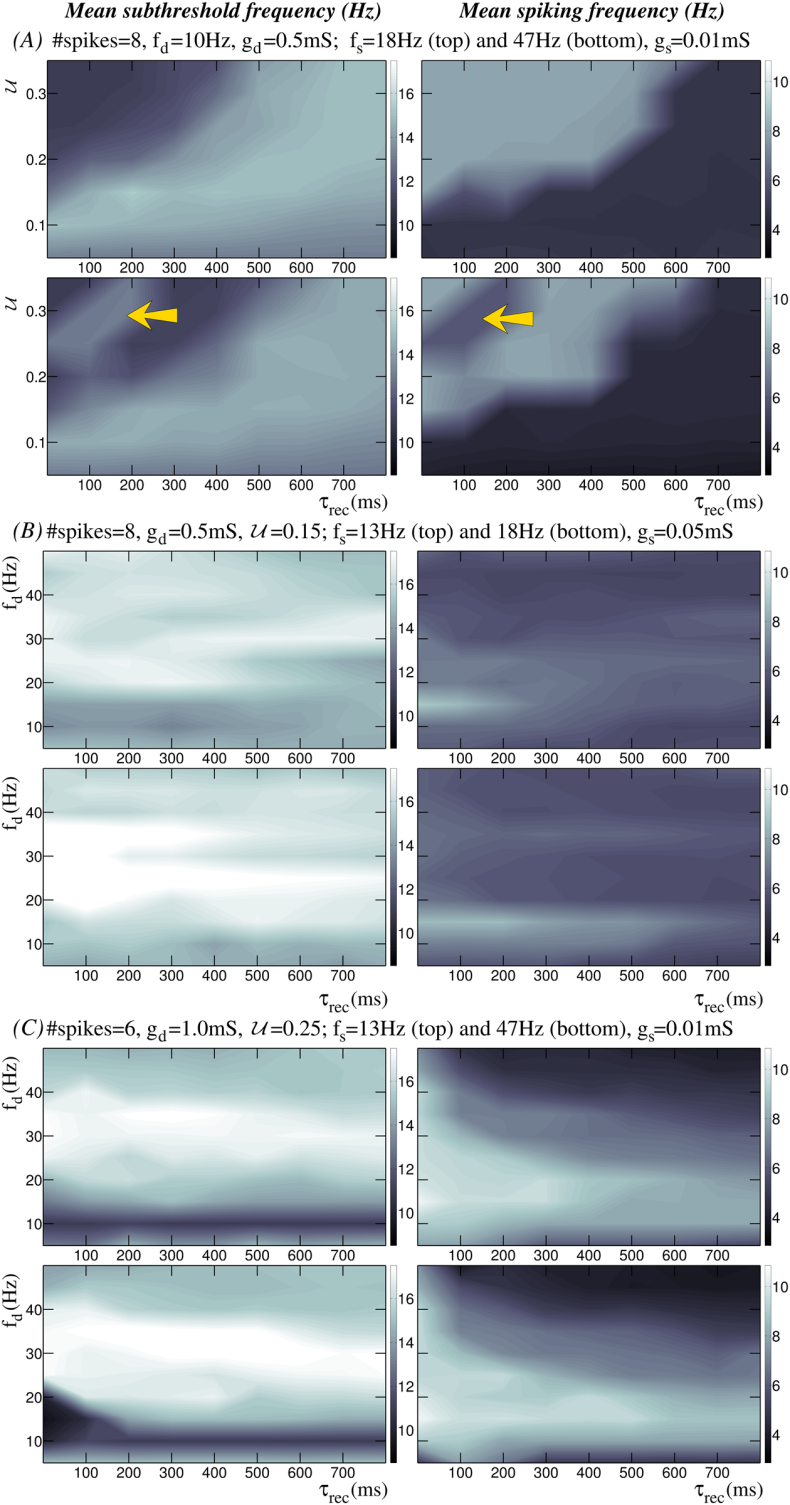
Input/output relations when the neuron receives an additional input in the context of the depression modulation. Panels display input/output activity maps equivalent to the maps shown in Fig 3 (#*spikes* denotes the number of spikes per burst and *f*_*d*_ the intraburst stimulation frequency through the dynamic channel), but when the neuron receives a second tonic incoming input with frequency *f*_*s*_ through the static channel.

Panels in Fig 7 show equivalent activity maps to those depicted in Fig 3, when the neuron receives a second tonic input through the static channel at different frequencies and with different synaptic conductance (*g*_*s*_ ≪ *g*_*d*_) in the context of the synaptic depression modulation. Again, we start by analyzing the dependence of the activity maps on the synaptic parameters shaping the depression level through the dynamic channel (Fig 7A). An additional 18*Hz*-stimulus through the static channel (cf. Fig 3A and top panels in Fig 7A) produces a general increase in the subthreshold oscillation frequency, except when U≥0.25 and *τ*_*rec*_ ≤ 200*ms*; and only a slight increase in the spiking activity in those regions with a low spiking response, while in the regions with a high spiking response the output spiking frequency is nearly the same. However, if the frequency of the additional stimulus is 47*Hz* instead of 18*Hz* (Fig 7A, bottom panels), although the additional input is a high-frequency excitatory stimulus, the resulting spiking activity decreases (as compared to the 18*Hz*-stimulation case) and, a new input/output relationship appears for low values of *τ*_*rec*_ and high values of U (see the region indicated by the arrow). In fact, in this region the spiking frequency drops, mainly due to failure in spike generation in response to incoming action potentials, and the subthreshold oscillation frequency increases because of the birth of fast oscillations which are not present in the intrinsic neuron dynamics. None of these responses correspond to the expected behavior of the neuron receiving a static excitatory stimulus at high frequency (cf. Fig 1B). Therefore, the emergence of this new input/output preference under the effect of synaptic depression reflects the complexity of the resonant processes involved in the computation of the neuron response. Panels B and C in Fig 7 illustrate that the input/output preferences arising from the effect of the synaptic depression can be modified when an additional static synaptic input is received by the neuron. The dynamic synapse modulates the intrinsic excitability of the cell and thus the response to stimuli through the static channel varies as a function of this modulation. For example, a tonic input with a frequency of 13*Hz* (top panels) can eliminate some of the preferences observed in Fig 3B. Note that even a stimulus delivered through a weak static channel (cf. Fig 7C where *g*_*s*_ = 0.01*mS*) can tune the postsynaptic response allowing specific input/output relationships to emerge. Fig 7 also shows that large values of synaptic conductance can diminish the modulatory influence of synaptic depression.

### Synaptic depression facilitates detection of stimuli delivered through weak static synaptic channel

The changes in subthreshold oscillation amplitude and frequency in the postsynaptic neuron described in the previous sections—which are induced by the short-term synaptic plasticity—can have strong computational implications during the processing of relevant information encoded in signals arriving through other synapses to the same neuron. With this in mind, we have investigated the conditions in which the postsynaptic neuron can efficiently detect specific frequencies in stimuli received through an additional synaptic channel. On one hand, the higher the amplitude of the subthreshold oscillations, the lower the synaptic current required to reach the firing threshold. On the other hand, the faster the subthreshold oscillation (e.g., due to changes produced in the subthreshold frequency during the stimulation periods), the larger the probability to respond to a synaptic input at a maximum depolarization.

**Fig 8:**
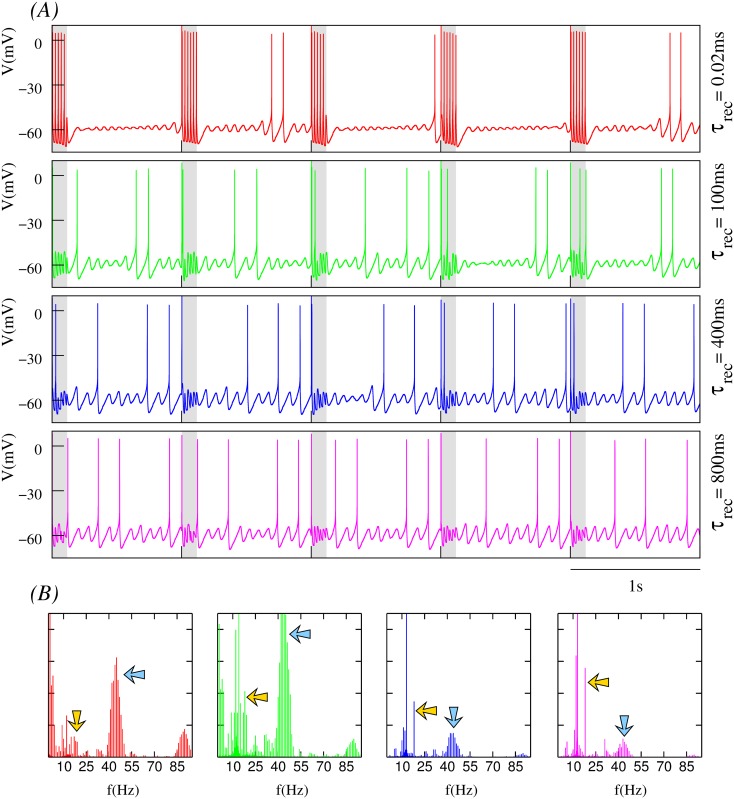
Synaptic depression facilitates the detection of weak synaptic currents delivered through a static channel. The neuron dynamics is modulated by the high-frequency dynamic input described in Fig 4—i.e., a bursting signal with 6 spikes per burst delivered at an intraburst spiking frequency of 45*Hz* (the corresponding frequency peaks are pointed out by blue arrows in the power spectra) through a dynamic synapse with *g*_*d*_ = 1.0*mS* and U=0.25. In addition to this input through the dynamic synapse, the neuron now receives an additional stimulus through a static synapse with *g*_*s*_ = 0.01*mS*. The input received through this weak synapse corresponds to a 18*Hz* tonic spiking signal (yellow arrows in the power spectra). Time series in panel A are equivalent to those shown in Fig 4A. Note how the additional stimulation frequency focuses the power spectra shown in panel B as the depression level increases.

**Fig 9:**
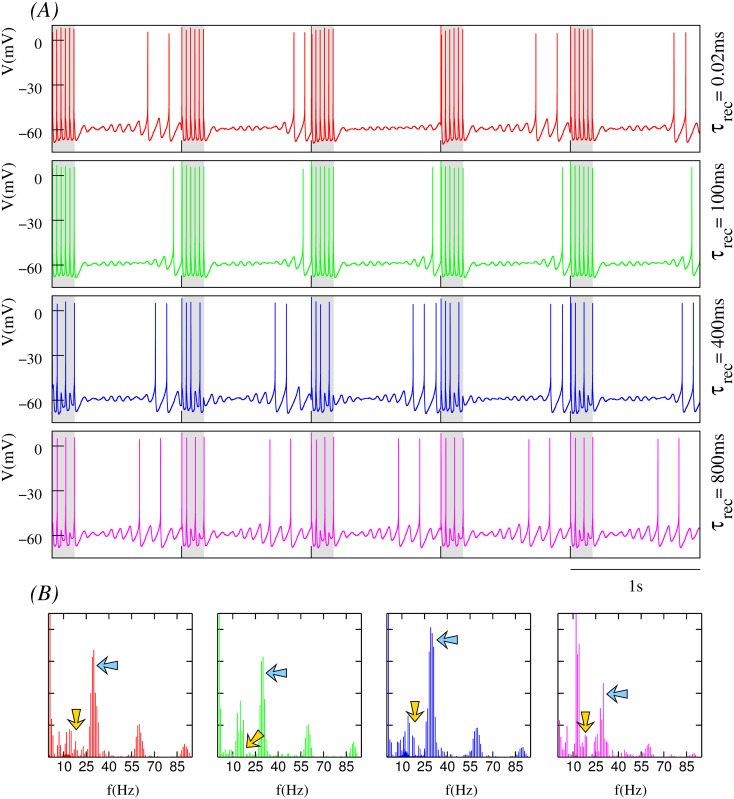
Processing of an additional stimulus in the context of the modulation induced by an intermediate frequency stimulus delivered through a dynamic synapse. This figure is equivalent to Fig 8 but now the neuron dynamics is modulated by the dynamic input described in Fig 5—i.e., a bursting signal with 6 spikes per burst delivered at an intraburst spiking frequency of 30*Hz* through a dynamic synapse with *g*_*d*_ = 1.0*mS* and U=0.25.

**Fig 10:**
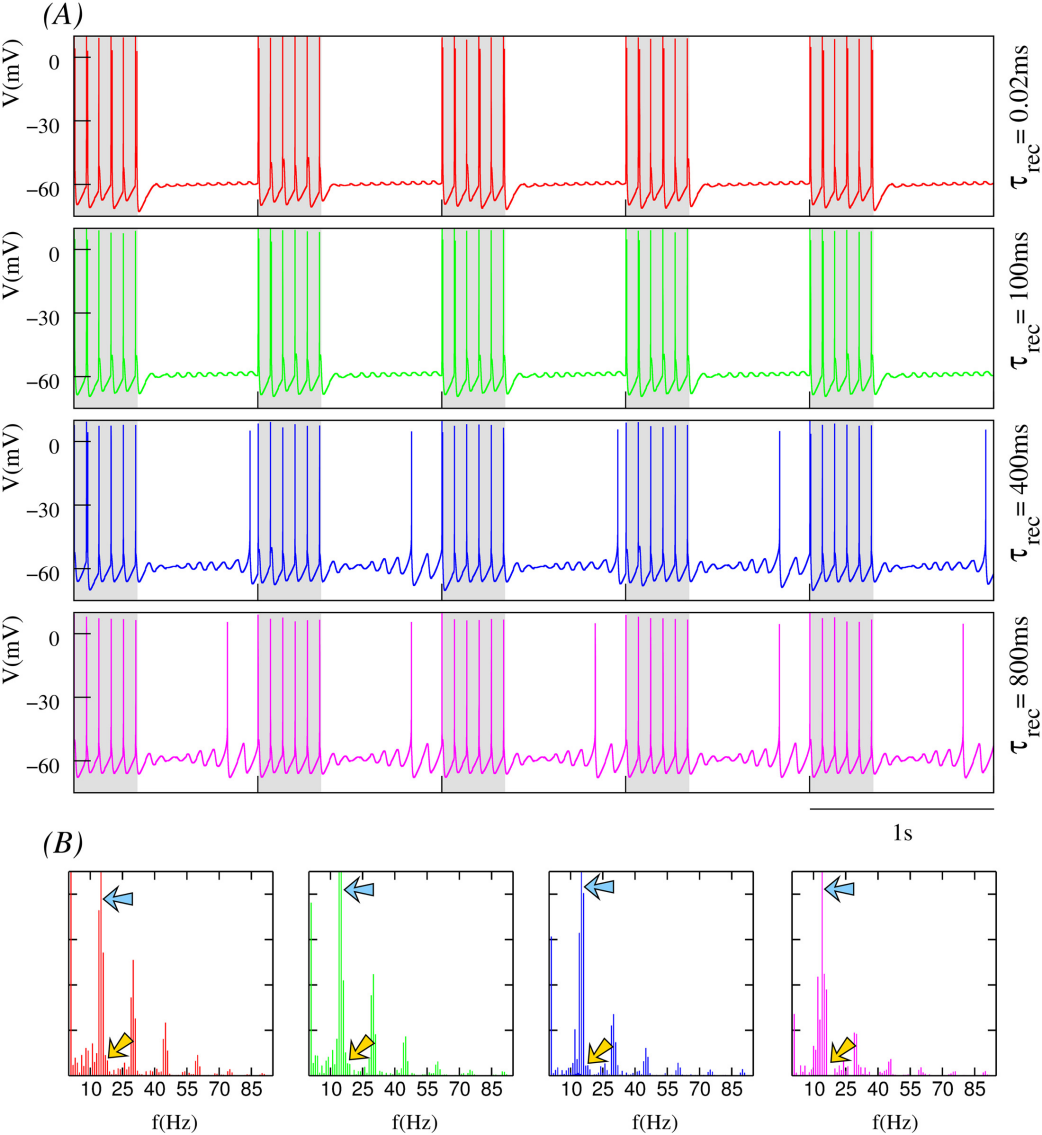
Additional stimuli delivered through a weak synapse are not detected under the modulation of low intraburst stimulation frequencies. This figure is equivalent to Figs 8 and 9, but now the dynamic input is the one shown in Fig 6 (a sequence of bursts with 6 spikes each, an intraburst spiking frequency of 15*Hz*, *g*_*d*_ = 1.0*mS* and U=0.25). In this case, due to the low stimulation frequency, there is no significant effect of synaptic depression and the additional static stimuli at 18*Hz* is not detected.

Figs 8, 9 and 10 illustrate the main findings obtained in out analysis. More precisely, the figures depict how the postsynaptic neuron—receiving bursts of spikes through a dynamic synapse with different levels of depression—is able to detect an additional periodic stimulus set at a frequency, for instance, of 18*Hz* arriving through a second synaptic channel without short-term synaptic plasticity. Each of these figures show the corresponding neuron time series (panel A) and power spectra (panel B) for the cases of high, medium and low depression levels in the dynamic synapse channel discussed in Figs 4, 5 and 6. In the power spectra we observe that the neuron detects the additional stimulus, frequency pointed out by the yellow arrow, in the cases where the depression effect is significant (e.g., simulations with large values of *τ*_*rec*_ in Fig 4). To corroborate the results derived from the power spectra, we have calculated the signal-to-noise ratio (SNR) of the output signals. The SNR is estimated using the definition provided in [63] as the ratio of input signal power to the mean spectral density power in a narrow window around the input signal frequency. Panel A in S3 Fig shows this analysis for the situations illustrated in Figs 4, 5 and 6. Note that SNR increases with *τ*_*rec*_ for relatively large intraburst frequencies in the input signal where depression has a significant effect. The increasing trend of SNR values corresponds to the detection of the additional 18*Hz*-input through the static synapse in the context of the depression modulation. In all cases discussed above, we set *g*_*s*_ = 0.01*mS* which means that the detection occurs even when the additional stimulus is delivered through a weak synapse. On the other hand, when the neuron only receives this periodic stimulus, the resulting neuron dynamics is the same as that for an isolated neuron and no detection occurs as shown in the cyan trace in panel A of S3 Fig.

### Input discrimination and generation of new information

The activity maps analyzed above allow the identification of regions in the parameter space, i.e., combinations of specific stimulation frequencies and dynamic synapse parameters, in which the interplay between the characteristic time scale of intrinsic oscillations and of the synaptically modulated input generates several complex input/output relations. It is important to note that these relations can result in channel-specific information discrimination mechanisms allowing single neurons to build input source and context dependent responses by tuning the dynamic channel parameters. In this section, we analyze how some of the relations arising from the combination of dynamic synapses and intrinsic subthreshold oscillations could be associated to discrimination processes and/or to the generation of new information. Note that in the examples discussed below the strength of the dynamic synapse (*g*_*d*_) is lower than in the cases previously discussed in Figs 4–6 (0.5*mS* vs. 1.0*mS*).

**Fig 11:**
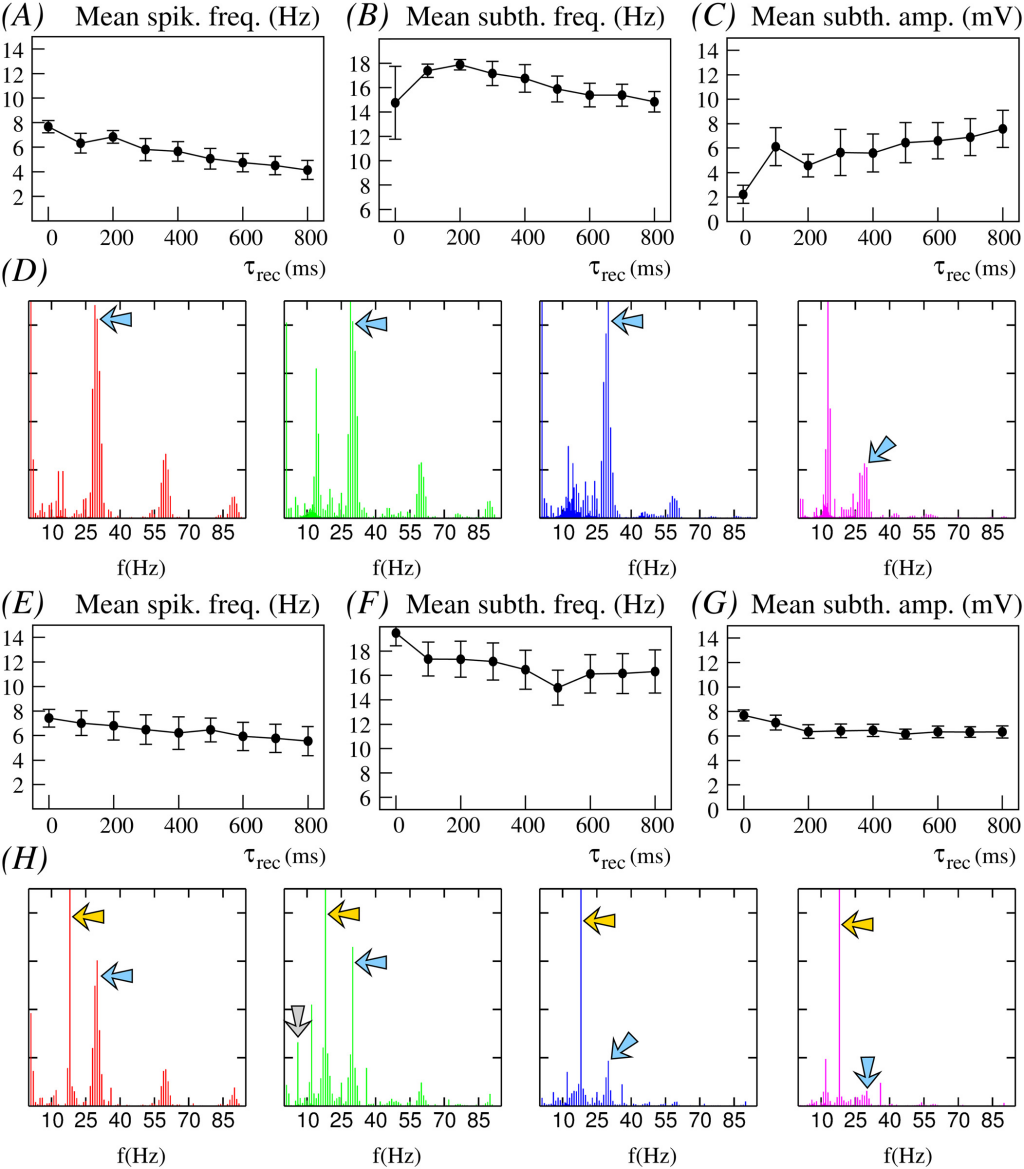
Example of input discrimination and generation of new information. (A-C) Dependence of the mean spiking frequency, the mean subthreshold frequency and the mean subthreshold amplitude of the postsynaptic neuron on the depression level as given by *τ*_*rec*_. The incoming stimuli consists of 8-spike bursts with a 30*Hz* intraburst spiking frequency (blue arrows in the power spectra) delivered through a dynamic synapse with *g*_*d*_ = 0.5*mS* and U=0.35. (D) Normalized power spectra corresponding to different depression levels in the simulations shown in panels A-C: *τ*_*rec*_ = 0.02*ms* (red), *τ*_*rec*_ = 100*ms* (green), *τ*_*rec*_ = 400*ms* (blue) and *τ*_*rec*_ = 800*ms* (magenta). (E-G) Response of the same neuron of panels A-C, but when an additional tonic spiking signal at 18*Hz* (yellow arrows in the power spectra) arrives through the static synapse with *g*_*s*_ = 0.05*mS*. (H) Normalized power spectra corresponding to different depression levels in the simulations shown in panels E-G: *τ*_*rec*_ = 0.02*ms* (red), *τ*_*rec*_ = 100*ms* (green), *τ*_*rec*_ = 400*ms* (blue) and *τ*_*rec*_ = 800*ms* (magenta). The gray arrow in the green power spectrum denotes a new output rhythm not related to frequencies present in the input nor in the intrinsic dynamics. Time series corresponding to the data shown in this figure are plotted in S4 Fig.

**Fig 12:**
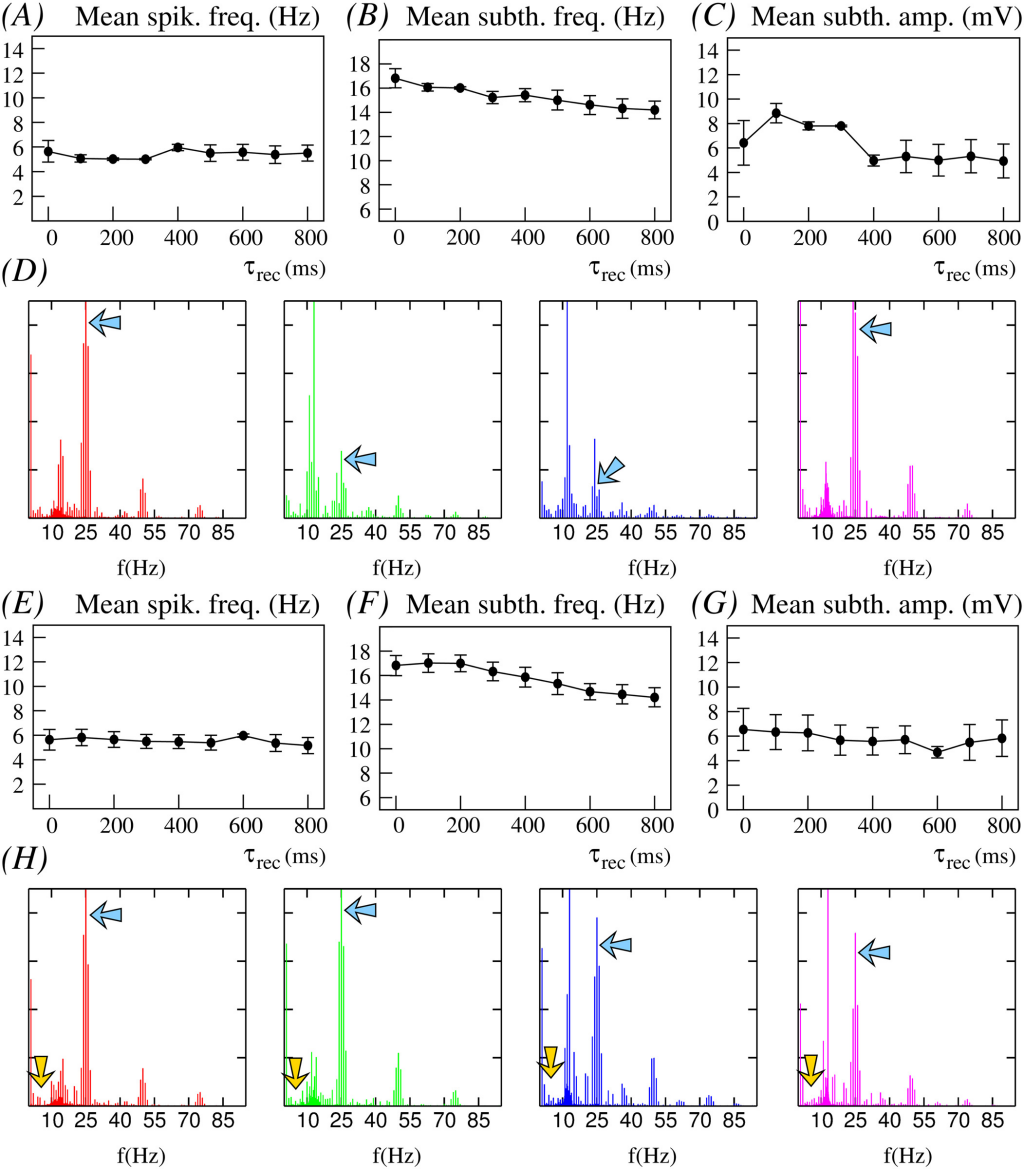
Synaptic depression can tune the resonant properties of the neuron by means of changes in the subthreshold amplitude. Figure equivalent to Fig 11 for a 25*Hz* spiking frequency input delivered through a dynamic synapse with *g*_*d*_ = 0.5*mS* and U=0.15. Power spectra correspond to different depression levels: *τ*_*rec*_ = 0.02*ms* (red), *τ*_*rec*_ = 100*ms* (green), *τ*_*rec*_ = 400*ms* (blue) and *τ*_*rec*_ = 800*ms* (magenta). In panel B, the frequency of the additional spiking stimulus is 5*Hz* and it is received through a static synapse with *g*_*s*_ = 0.01*mS*. Time series corresponding to the data shown in this figure are plotted in S5 Fig.

The first example is shown in Fig 11 (see also S4 Fig for time series) and corresponds to simulations with a 30*Hz* intraburst stimulation frequency (the same as in Fig 5). A simple inspection of the time series depicted in panels A of Fig 5 and S4 Fig (note that they are equivalent in terms of depression level and intraburst stimulation frequency) shows that significant changes appear now in the subthreshold activity for relative low values of *τ*_*rec*_ (cf. Figs 5C and 11B). As we have discussed above, the general trend observed in our simulations is that the higher the depression level, the lower the level of subthreshold activity in the postsynaptic cell. However, in this example we would like to highlight the situations where *τ*_*rec*_ = 100*ms* and *τ*_*rec*_ = 200*ms*. For these depression levels, the amplitude of the subthreshold oscillations does not diminish as in the general case (Fig 5), but synaptic input is invested in potentiate the oscillatory activity (panel A in S4 Fig). Note, for example, how the mean subthreshold frequency is now maximum for *τ*_*rec*_ = 100*ms* and the corresponding mean amplitude is also larger (cf. Figs 11B and 5C). Changes in the subthreshold frequency and amplitude have a significant effect in the resonant properties of the neuron for these values of short-term synaptic depression. Looking at the power spectra depicted in Fig 11D, when *τ*_*rec*_ = 100*ms* (green traces), in contrast to the equivalent power spectrum in Fig 5E, we clearly distinguish the intrinsic subthreshold oscillation frequency. These power spectra panels also show that for *τ*_*rec*_ = 800*ms* the intrinsic subthreshold frequency prevails over the intraburst stimulation frequency. These results reveal how an individual neuron can generate very different responses to a given stimulus depending on its current intrinsic excitability and the specific properties of the depressing synaptic channel, which allows the implementation of synaptic-dependent discrimination mechanisms at the single neuron level. If now the neuron receives an additional static stimulus at 18*Hz* (Fig 11, panels E-H), we observe that its response in terms of mean spiking frequency and mean subthreshold frequency is not affected. Only in the case of a synapse with almost no depression (*τ*_*rec*_ = 0.02), there is a significant change in the mean subthreshold frequency. The additional input current seems to be mainly employed to regularize the subthreshold oscillation amplitude. Note that with the additional stimulus, the variability of the subthreshold amplitude diminishes and its mean value is nearly the same for all depression levels. The power spectra represented in Fig 11H show that the neuron now is able to detect the 18*Hz*-stimulus independently of the depression level (note that in this case *g*_*s*_ = 0.05*mS*). The SNR analysis corroborates this result (see panel B in S3 Fig). On the other hand, this example illustrates the generation of new information due to synaptically modulated input/output transformation. This is observed in the form of a peak frequency component between 5 and 6*Hz* in the power spectrum corresponding to *τ*_*rec*_ = 100*ms* (peak indicated by the arrow in the green trace in Fig 11H). This new output rhythm appears only for this value of *τ*_*rec*_ and reflects a non-trivial input-output relationship consequence of the complex interaction among the time scales of the different dynamics shaping the neuron response. Note that most power spectra shown in Figs 4–6 and 8–12 include peaks not related to frequencies present in the input or in the intrinsic dynamics.

Another example of synaptic-modulated discrimination is depicted in Fig 12 and its corresponding time series depicted in S5 Fig. Here we observe that for *τ*_*rec*_ values ranging from 100 to 500*ms* the neuron response is highly stereotyped in such a way that it generates a nearly periodic activity (e.g., see green and blue time series: the response is exactly the same in the five displayed stimulation cycles). In these cases, the neuron spiking frequency is not influenced by the degree of depression (note that the mean spiking frequency is nearly the same for all depression levels). This is mainly due to failure in the generation of postsynaptic spikes during the stimulation period regardless of the degree of depression. Interestingly, this is an example where the relationship between the level of depression and the subthreshold oscillation amplitude changes compared with the cases described in previous sections, and there is not a general monotonic increase of the mean subthreshold oscillation amplitude with the degree of depression. This is illustrated in Fig 12C, where it is shown that the amplitude is higher for small values of *τ*_*rec*_ and decreases as the level of depression grows. This makes (i) the intrinsic oscillation frequency prevail over the stimulation frequency for small values of *τ*_*rec*_ (e.g. *τ*_*rec*_ = 100*ms*), and (ii) the power spectra with high depression levels be equivalent to the ones with a nearly static synapse. On the other hand, data shown in panels C and G of Fig 12 illustrate how a stimulus arriving through a weak static synapse—not affecting the neuron behavior in the absence of other stimuli—can tune the subthreshold amplitude and change the resonant properties of the neuron when an input modulated by synaptic depression is also present (cf. power spectra in Fig 12D and 12H). Again, this last result emphasizes the relevance of the interplay between the precise timings of subthreshold oscillations and dynamic synapses.

**Fig 13:**
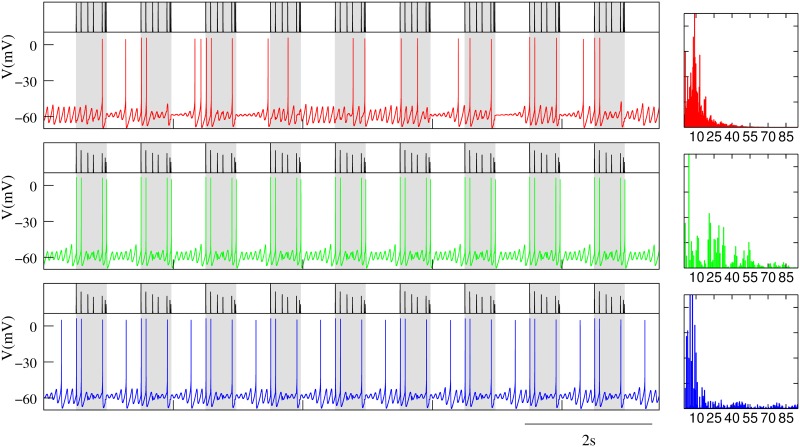
Synaptic-dependent discrimination of input patterns. The dynamics of intrinsic oscillations can be combined with the characteristic time scale of the modulatory input received through a depressing synapse to detect specific input patterns and generate different stereotyped outputs in response. The figure shows the response of a neuron to a pattern delivered through input channels with different depression levels. Plots in the right column correspond to the normalized power spectra of the output signal. In all cases *g*_*d*_ = 0.32*mS* and U=0.41, while the depression level is defined by the value of *τ*_*rec*_: 0.02*ms* in the top panel (i.e., a static synapse), 400*ms* in the middle panel, and 800*ms* in the bottom panel. The input pattern consists of seven spikes with the following timing distribution: *ISI*_1–2_ = 75*ms*, *ISI*_2–3_ = 105*ms*, *ISI*_3–4_ = 85*ms*, *ISI*_4–5_ = 136*ms*, *ISI*_5–6_ = 46*ms* and *ISI*_6–7_ = 15*ms*. Black trace in each panel plots the evolution of the corresponding fraction of bound receptors in the synaptic cleft. Short-term synaptic depression leads to specific stereotyped responses to this input. When *τ*_*rec*_ is in the range 325–550*ms* (middle panel), the output consists of four spike: two at the beginning of the input pattern presentation and two at the end. In the range 575–800*ms* (bottom panel), the neuron generates two spikes at the beginning of the input pattern, one at the end and another output spike four oscillations after the end of the stimulation. However, when the pattern arrives through a static synapse or a synapse with lower value of *τ*_*rec*_, the response is not regular (note in the top panel the different responses to the pattern and the different subthreshold oscillation regimes after the stimulation).

The discrimination mechanism discussed above allows the detection and distinct processing of input patterns by tuning cost-effective channel parameters that induce a different input/output transformation in the cell. Fig 13 shows the different responses of a neuron to a given 7-spike input pattern as a function of the depression level. In contrast to the results presented so far, in this example, synaptic parameters are tuned to maximize the neuron’s resonant response to the incoming pattern (cf. Fig 1 where action potentials are delivered at the peak of the subthreshold depolarization). When the input pattern is received through a depressing synapse (middle and bottom panels), the resonant properties arising from the interplay between subthreshold oscillations and the dynamics of the dynamic synapse make the neuron respond to the incoming input in a stereotyped mode (note that this stereotyped output can change as a function of the level of depression). However, when the pattern is delivered through a static synapse (top panel, note the total input current received by the postsynaptic neuron is larger than the current received through the depressing synapse), the neuron generates a non-regular response and its firing rate is lower. Panels on the right show richer power spectra as compared to those presented in previous figures.

## Discussion

Subthreshold oscillations are observed in many neuron types [2, 13, 64, 65]. Synaptic depression and facilitation are a form of short-term plasticity exhibited by a wide variety of synapses in the nervous system [24]. Although both subthreshold oscillations and dynamic synapses have been extensively studied both experimentally and theoretically, their joint action on single neuron dynamics has attracted less attention. In this paper, we have used a single neuron conductance-based model with a dynamic synaptic channel to show that intrinsic subthreshold activity can be effectively modulated by synaptic depression. This modulation affects the neuron’s excitability and its input/output relationships by reshaping the resonant properties arising from the subthreshold oscillations.

The hyperpolarization level of the postsynaptic neuron, the amplitude and shape of its subthreshold oscillations or the resulting firing threshold are all factors modulated by the dynamic synapse, which in turn build up new resonances that influence the response to other incoming stimuli. Thus, dynamic synapses can affect both the subthreshold and the spiking dynamics of the postsynaptic neuron in a channel specific manner. This influence depends on the temporal structure of the stimulus, which is relevant for a stimulus history-dependent processing. For simplicity, in our study we have used a specific set of burst trains as input information. Specific temporal structures in the input can reveal specific input/output preferences.

The role of neuron and synaptic heterogeneity has been addressed in several works (e.g., see [12, 60–62]). To what extent can dynamic synapses allow an efficient tuning of neuron function beyond or in addition to intrinsic cell dynamics? The results presented in this paper indicate that synaptic depression could largely contribute to shape preferred input/output relationships in individual neurons. Building these relationships from the combination of intrinsic subthreshold oscillations and dynamic synapses can make them specific of individual synaptic channels, and thus allow flexibility regarding when or from whom (i.e., in response to which presynaptic origin) this distinct processing takes place. These information discrimination mechanisms can be built in single neurons by tuning cost-effective channel parameters without altering key parameters of intrinsic neuronal dynamics. A specific tuning in the depression of a single synaptic channel may underlie the specific function of the input/output transformation in the cell. This can contribute to the context-specific processing and multifunctional nature of several neurons and neural networks in the nervous system. On the other hand, synaptic modulation of subthreshold oscillations could balance or regulate heterogeneous neuron dynamics when heterogeneity is not a desirable property related to neural function, but an avoidable departing point to deal with.

Although not addressed in this paper, the interaction between subthreshold oscillations and dynamic synapses can also give rise to collective phenomena in network dynamics. For example, in the inferior olive, a system that has been proposed as a generator of timing signals in the cerebellum and as a coordinator of different rhythms through the intrinsic oscillatory properties of the olivary neurons and their electrical interconnections [7, 66], dynamic synapses could highly influence the way the spatio-temporal patterns are generated and propagated throughout this network [18]. Similarly, in other systems the preferred input/output relationships that arise from the combination of subthreshold oscillations and dynamic synapses may change or enhance specific functions associated with these systems. Here, we have shown that the combined effect of dynamic synapses and subthreshold oscillations can boost the recognition of signals arriving through weak synapses. The effect of short-term synaptic plasticity on the stability of localized activity patterns, or bumps, and on the emergence of spatio-temporal patterns of neural activity, such as traveling spots and traveling waves, has been reported in [67–69]. These studies suggest that relevant phenomena appear as a consequence of the instabilities induced by the interplay between the underlying noise and synaptic depression, which could also be discussed in a context where intrinsic subthreshold oscillations are present. In spite of its subthreshold nature, the information encoded in these oscillations could be efficiently processed by dynamic synapses at different noise levels through stochastic resonance mechanisms [53–55].

In this paper we have focused on the effect of rhythmic inputs such as those present in the cerebellar systems. Our study could be generalized to other activation patterns, e.g. Poisson like. On the other hand, the effect of synaptic fluctuations can also be considered, as they have been shown to influence the neuron’s response in combination with the depression level [70]. Beyond the interaction with synaptic depression dynamics, subthreshold oscillations can also interact with other synaptic mechanisms such as short-term synaptic facilitation, STDP [71] and other plasticity mechanisms that can influence intrinsic and network properties, also in the context of inhibitory synapses. The results reported in this paper call for further experimental and theoretical studies in this direction.

## Supporting Information

S1 Fig Short-term synaptic plasticity mechanisms in the proposed synaptic model. The figure shows the evolving dynamics of the fraction of bound receptors in the synaptic cleft, *r*(*t*), when two tonic spiking stimuli at different frequencies (20*Hz* and 35*Hz* in panels A and B, respectively) are transmitted through a static—i.e., a synapse with no dynamical synaptic processes, neither depression nor facilitation—(gray traces), a depressing (magenta traces) and a facilitating (blue traces) synapse. Note that for the static synapse *r* reaches the same peak value with each presynaptic action potential independently of the stimulation frequency. In contrast, in the depressing synapse, *r* drops with each action potential until it reaches a stable value. This value depends on the stimulation frequency. In the case of facilitating synapses, the opposite occurs and the fraction of bound receptors increases with each action potential.
(TIF)

S2 FigThe interaction between intrinsic oscillations and synaptically modulated input can lead to complex preferred input/output relations.The figure shows the different response of a neuron to the same input delivered through a dynamic synapse with *g*_*d*_ = 0.5*mS* and U=0.15 as a function of the depression level as given by *τ*_*rec*_. The incoming stimulus consists of 8-spike bursts, with an interburst frequency of 1*Hz* and an intraburst spiking frequency of 15*Hz* (see activity maps in Fig 3B in the main text). (A-C) Dependence of the mean spiking frequency, the mean subthreshold frequency and the mean subthreshold amplitude of the postsynaptic neuron on the short-term synaptic depression. These panels illustrate how different factors underlying the neuron’s resonant properties can be modulated by synaptic depression. (D) Normalized power spectra corresponding to different depression levels in the simulations whose data are plotted in panels A-C: *τ*_*rec*_ = 0.02*ms* (red), *τ*_*rec*_ = 300*ms* (green), *τ*_*rec*_ = 500*ms* (blue) and *τ*_*rec*_ = 800*ms* (magenta). Blue arrows identify the peak frequency components corresponding to the intraburst stimulation frequency. Although the main frequency component is the same, synaptic depression modulates the amplitude and frequency of the oscillations. This produces different subthreshold and spiking activity modes, which can implement complex preferred input/output relations beyond simple resonant responses.(TIF)

S3 FigSignal-to-noise ratio (SNR) at 18*Hz* when the neuron receives a tonic input at this frequency through a weak static synapse under the modulatory effect of different depressing inputs.The SNR is estimated as the ratio of input signal power (in this case 18*Hz*) to the mean spectral density power around the input frequency (18 ± 1.5*Hz*). Left panel: The neuron dynamics is modulated by a bursting signal with 6 spikes per burst delivered at an intraburst spiking frequency of 45*Hz* (blue trace), 30*Hz* (magenta) and 15*Hz* (brown) through the dynamic synapse with *g*_*d*_ = 1.0*mS* and U=0.25. These traces correspond to data plotted in Figs 8, 9 and 10 in the main text, respectively. Dashed line provides the reference value when the neuron only receives the static tonic input at 18*Hz*. In this situation no detection of the static stimulus occurs. Note how the SNR is higher in the cases where the depression effect is significant (see the increasing SNR trend in the blue and magenta traces as a function of *τ*_*rec*_). This points out that the neuron detects the additional stimulus at 18*Hz* in these cases. Right panel: The neuron dynamics is modulated by a bursting signal with 8 spikes per burst delivered at an intraburst spiking frequency of 30*Hz*. This trace corresponds to the data shown in Fig 11H in the main text and the time series depicted in S4 Fig. As in panel A, dashed line is the SNR when the neuron receives no dynamic input (note the increased *g*_*s*_ value, 0.05*mS* vs. 0.01*mS*). In this particular example, the combination of the time constants of intrinsic and synaptic dynamics potentiates the oscillatory activity increasing both the mean subthreshold frequency and amplitude. This boosts the resonant properties of the neuron and, as the high SNR for all *τ*_*rec*_ values indicates (cf. magenta trace in panel A), the 18*Hz* additional stimulus is easily detected independently of the depression level. For other intraburst stimulation frequencies, e.g., 15*Hz* or 45*Hz*, this specific input/output transformation does not appear and the neuron’s response is equivalent to that shown in panel A.(TIF)

S4 FigTime series corresponding to the power spectra shown in Fig 11 in the main text (color code is the same in both figures).Panel A: Only the dynamic channel is active with *g*_*d*_ = 0.5*mS* and U=0.35. The incoming stimulus consists of bursts with 8 spikes with a spiking frequency equal to 30*Hz*. Each time series correspond to a different depression level: *τ*_*rec*_ = 0.02*ms* (red), *τ*_*rec*_ = 100*ms* (green), *τ*_*rec*_ = 400*ms* (blue) and *τ*_*rec*_ = 800*ms* (magenta). Panel B: Equivalent time series when an additional tonic stimulus at 18*Hz* arrives through the static channel with *g*_*s*_ = 0.05*mS*.(TIF)

S5 FigTime series corresponding to the power spectra shown in Fig 12 in the main text (color code is the same in both figures).Panel A: The incoming stimulus consists of 8-spike bursts with a spiking frequency equal to 25*Hz* received through a dynamic synapse where *g*_*d*_ = 0.5*mS* and U=0.15. Each time series correspond to a different depression level: *τ*_*rec*_ = 0.02*ms* (red), *τ*_*rec*_ = 100*ms* (green), *τ*_*rec*_ = 400*ms* (blue) and *τ*_*rec*_ = 800*ms* (magenta). Panel B: Equivalent time series when an additional tonic stimulus at 5*Hz* arrives through the static channel with *g*_*s*_ = 0.01*mS*.(TIF)
