# Supplementary figures and images for: Interplay between Subthreshold Oscillations and Depressing Synapses in Single Neurons
Source: PLoS One. 2016 Jan 5;11(1):e0145830. doi: 10.1371/journal.pone.0145830 (PMC4701431; doi:10.1371/journal.pone.0145830)

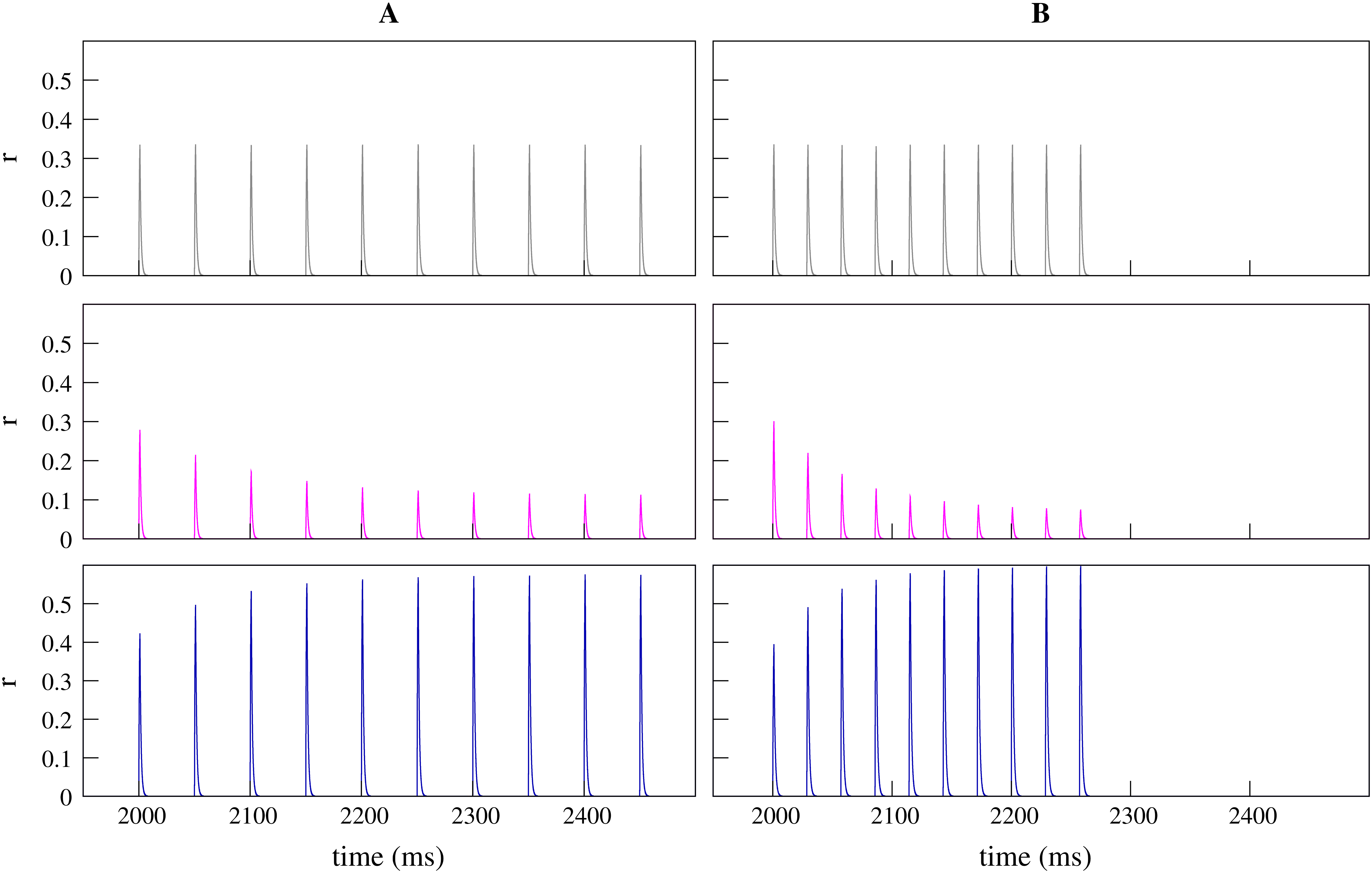

Supplement: S1 Fig — The figure shows the evolving dynamics of the fraction of bound receptors in the synaptic cleft, r(t), when two tonic spiking stimuli at different frequencies (20Hz and 35Hz in panels A and B, respectively) are transmitted through a static—i.e., a synapse with no dynamical synaptic processes, neither depression nor facilitation—(gray traces), a depressing (magenta traces) and a facilitating (blue traces) synapse. Note that for the static synapse r reaches the same peak value with each presynaptic action potential independently of the stimulation frequency. In contrast, in the depressing synapse, r drops with each action potential until it reaches a stable value. This value depends on the stimulation frequency. In the case of facilitating synapses, the opposite occurs and the fraction of bound receptors increases with each action potential. (TIF) [file pone.0145830.s001.tif]

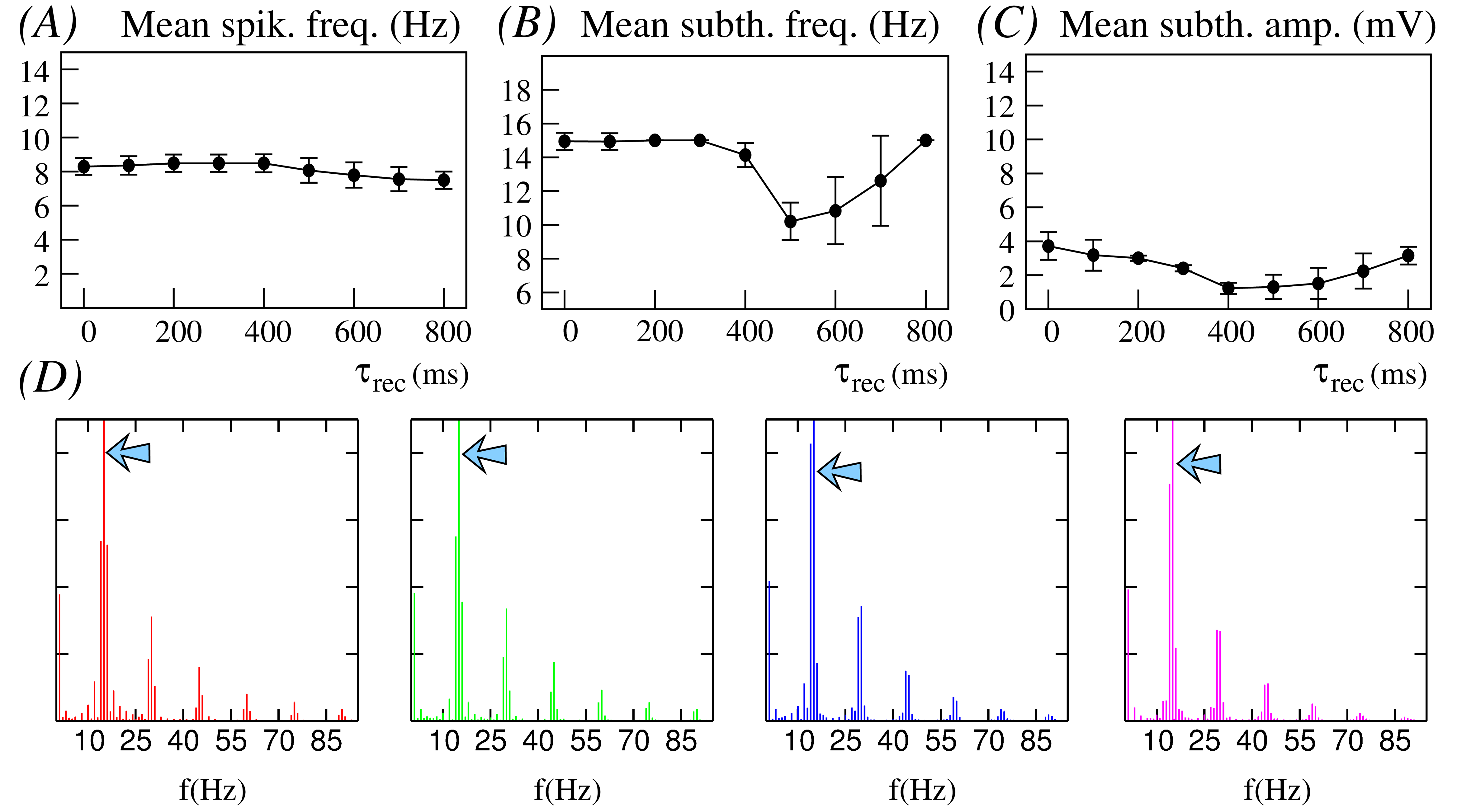

Supplement: S2 Fig — The figure shows the different response of a neuron to the same input delivered through a dynamic synapse with gd = 0.5mS and U=0.15 as a function of the depression level as given by τrec. The incoming stimulus consists of 8-spike bursts, with an interburst frequency of 1Hz and an intraburst spiking frequency of 15Hz (see activity maps in Fig 3B in the main text). (A-C) Dependence of the mean spiking frequency, the mean subthreshold frequency and the mean subthreshold amplitude of the postsynaptic neuron on the short-term synaptic depression. These panels illustrate how different factors underlying the neuron’s resonant properties can be modulated by synaptic depression. (D) Normalized power spectra corresponding to different depression levels in the simulations whose data are plotted in panels A-C: τrec = 0.02ms (red), τrec = 300ms (green), τrec = 500ms (blue) and τrec = 800ms (magenta). Blue arrows identify the peak frequency components corresponding to the intraburst stimulation frequency. Although the main frequency component is the same, synaptic depression modulates the amplitude and frequency of the oscillations. This produces different subthreshold and spiking activity modes, which can implement complex preferred input/output relations beyond simple resonant responses. (TIF) [file pone.0145830.s002.tif]

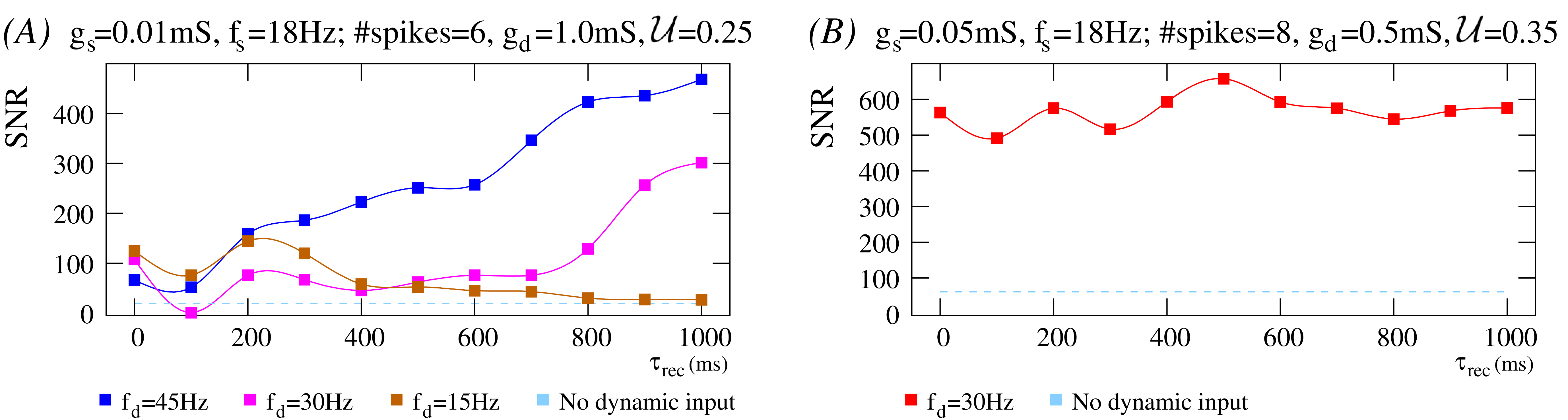

Supplement: S3 Fig — The SNR is estimated as the ratio of input signal power (in this case 18Hz) to the mean spectral density power around the input frequency (18 ± 1.5Hz). Left panel: The neuron dynamics is modulated by a bursting signal with 6 spikes per burst delivered at an intraburst spiking frequency of 45Hz (blue trace), 30Hz (magenta) and 15Hz (brown) through the dynamic synapse with gd = 1.0mS and U=0.25. These traces correspond to data plotted in Figs 8, 9 and 10 in the main text, respectively. Dashed line provides the reference value when the neuron only receives the static tonic input at 18Hz. In this situation no detection of the static stimulus occurs. Note how the SNR is higher in the cases where the depression effect is significant (see the increasing SNR trend in the blue and magenta traces as a function of τrec). This points out that the neuron detects the additional stimulus at 18Hz in these cases. Right panel: The neuron dynamics is modulated by a bursting signal with 8 spikes per burst delivered at an intraburst spiking frequency of 30Hz. This trace corresponds to the data shown in Fig 11H in the main text and the time series depicted in S4 Fig. As in panel A, dashed line is the SNR when the neuron receives no dynamic input (note the increased gs value, 0.05mS vs. 0.01mS). In this particular example, the combination of the time constants of intrinsic and synaptic dynamics potentiates the oscillatory activity increasing both the mean subthreshold frequency and amplitude. This boosts the resonant properties of the neuron and, as the high SNR for all τrec values indicates (cf. magenta trace in panel A), the 18Hz additional stimulus is easily detected independently of the depression level. For other intraburst stimulation frequencies, e.g., 15Hz or 45Hz, this specific input/output transformation does not appear and the neuron’s response is equivalent to that shown in panel A. (TIF) [file pone.0145830.s003.tif]

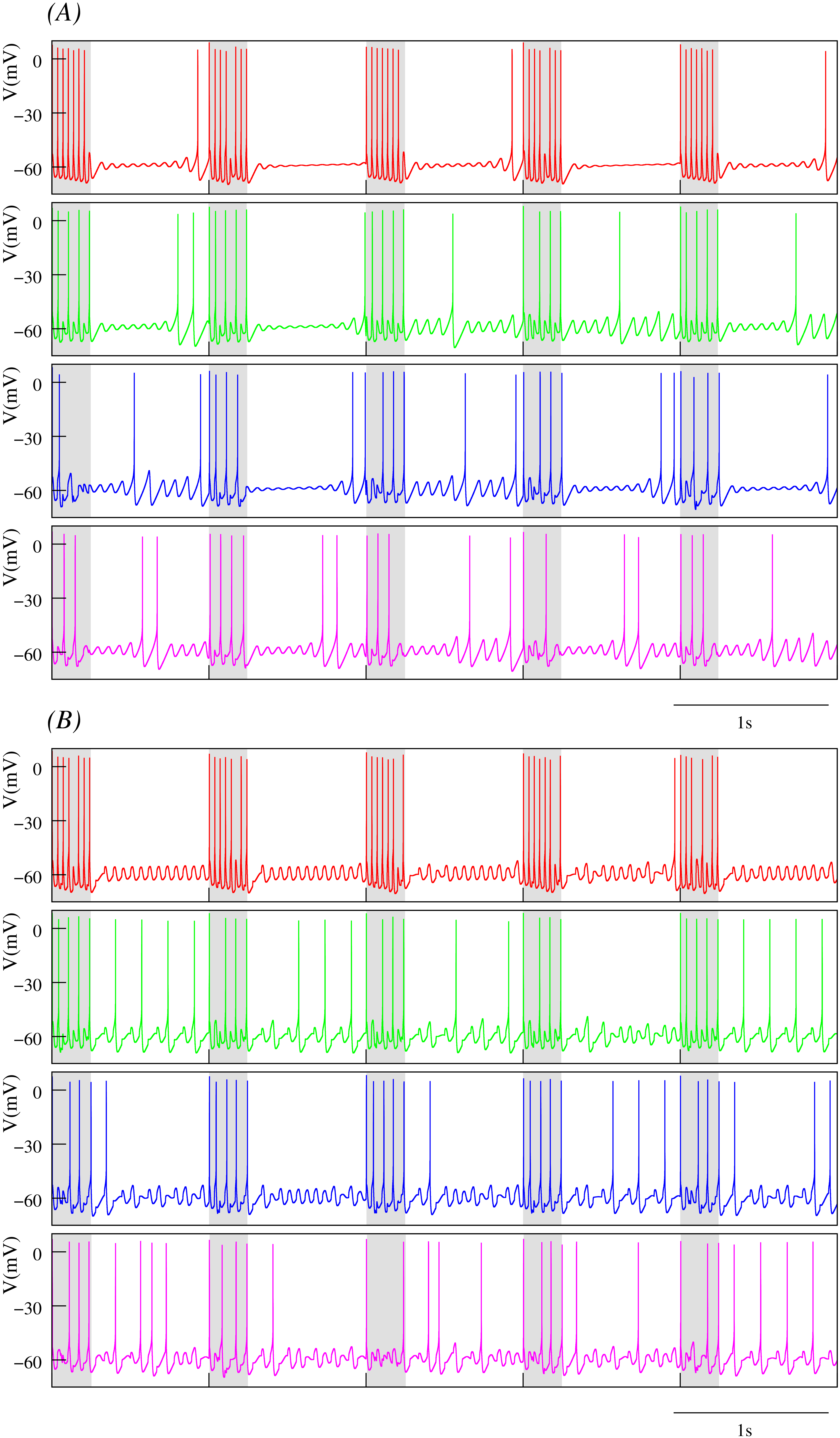

Supplement: S4 Fig — Panel A: Only the dynamic channel is active with gd = 0.5mS and U=0.35. The incoming stimulus consists of bursts with 8 spikes with a spiking frequency equal to 30Hz. Each time series correspond to a different depression level: τrec = 0.02ms (red), τrec = 100ms (green), τrec = 400ms (blue) and τrec = 800ms (magenta). Panel B: Equivalent time series when an additional tonic stimulus at 18Hz arrives through the static channel with gs = 0.05mS. (TIF) [file pone.0145830.s004.tif]

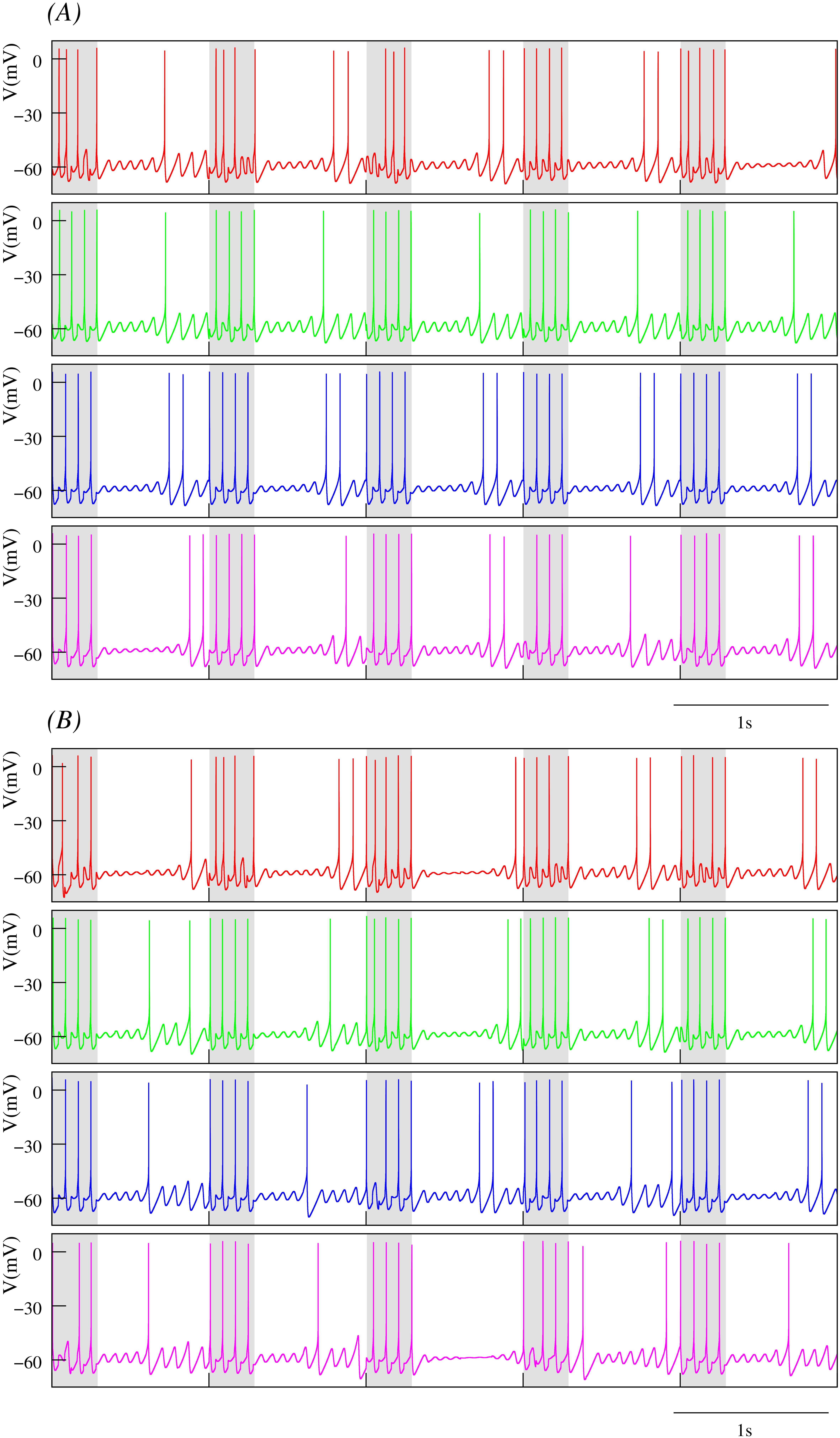

Supplement: S5 Fig — Panel A: The incoming stimulus consists of 8-spike bursts with a spiking frequency equal to 25Hz received through a dynamic synapse where gd = 0.5mS and U=0.15. Each time series correspond to a different depression level: τrec = 0.02ms (red), τrec = 100ms (green), τrec = 400ms (blue) and τrec = 800ms (magenta). Panel B: Equivalent time series when an additional tonic stimulus at 5Hz arrives through the static channel with gs = 0.01mS. (TIF) [file pone.0145830.s005.tif]
